# Mass spectrometry‐based abundance atlas of ABC transporters in human liver, gut, kidney, brain and skin

**DOI:** 10.1002/1873-3468.13982

**Published:** 2020-12-03

**Authors:** Zubida M. Al‐Majdoub, Brahim Achour, Narciso Couto, Martyn Howard, Yasmine Elmorsi, Daniel Scotcher, Sarah Alrubia, Eman El‐Khateeb, Areti‐Maria Vasilogianni, Noura Alohali, Sibylle Neuhoff, Lutz Schmitt, Amin Rostami‐Hodjegan, Jill Barber

**Affiliations:** ^1^ Centre for Applied Pharmacokinetic Research School of Health Sciences University of Manchester UK; ^2^ Clinical Pharmacy Department Faculty of Pharmacy Tanta University Egypt; ^3^ Pharmaceutical Chemistry Department College of Pharmacy King Saud University Riyadh Saudi Arabia; ^4^ Pharmaceutical Practice Department College of Pharmacy Princess Noura Bint Abdul Rahman University Riyadh Saudi Arabia; ^5^ Simcyp Division Certara UK Ltd Sheffield UK; ^6^ Institute of Biochemistry Heinrich Heine University Düsseldorf Germany

**Keywords:** ABC transporters, biliary atresia, global proteomics, human brain, human intestine, human kidney, human liver, human skin, mass spectrometry, total protein approach

## Abstract

ABC transporters (ATP‐binding cassette transporter) traffic drugs and their metabolites across membranes, making ABC transporter expression levels a key factor regulating local drug concentrations in different tissues and individuals. Yet, quantification of ABC transporters remains challenging because they are large and low‐abundance transmembrane proteins. Here, we analysed 200 samples of crude and membrane‐enriched fractions from human liver, kidney, intestine, brain microvessels and skin, by label‐free quantitative mass spectrometry. We identified 32 (out of 48) ABC transporters: ABCD3 was the most abundant in liver, whereas ABCA8, ABCB2/TAP1 and ABCE1 were detected in all tissues. Interestingly, this atlas unveiled that ABCB2/TAP1 may have TAP2‐independent functions in the brain and that biliary atresia (BA) and control livers have quite different ABC transporter profiles. We propose that meaningful biological information can be derived from a direct comparison of these data sets.

## Abbreviations

**ABC transporter**, ATP‐binding cassette transporter

**BCRP**, breast cancer resistance protein

**CNS**, central nervous system

**CSF**, cerebrospinal fluid

**FASP**, filter‐aided sample preparation

**LC‐MS**, liquid chromatography–mass spectrometry

**LSE**, living skin equivalent

**MDR**, multidrug resistance protein

**MRP**, multidrug resistance‐associated protein

**MS/MS**, tandem mass spectrometry

**PBPK model**, physiologically based pharmacokinetic model

**PCA**, principal component analysis

**P‐gp**, P‐glycoprotein

**PK**, pharmacokinetics

**QconCAT**, Quantification conCATemer

**TPA**, total protein approach

ATP‐binding cassette (ABC) transporters are a large and ubiquitous superfamily of transmembrane proteins that mediate ATP‐dependent transport of a wide range of endogenous and exogenous substances across biological membranes. ATP‐binding cassette transporters (ABC transporters) are involved in trafficking substrates across the cell membrane and also play an important role in intracellular compartmental transport and antigen presentation [1, 2]. The human genome encodes 48 ABC transporter genes expressed in various tissues (Table S1), with essential roles in many homeostatic physiological processes. Mutations in these genes are associated with diseases, including cystic fibrosis (ABCC7/CFTR) [3], Tangier disease (ABCA1/CERP) [4], Dubin–Johnson syndrome [ABCC2/multidrug resistance‐associated protein (MRP2)] [5] and gout [ABCG2/breast cancer resistance protein (BCRP)] [6]. Variations in expression patterns (mainly in ABCB1/multidrug resistance protein (MDR1), ABCG2/BCRP and ABCCs/MRPs) have been linked to toxicity and resistance to therapeutic agents, particularly in cancer chemotherapy [7, 8].

In pharmacology, transporters play a key role in trafficking drugs and their metabolites across membranes; their expression patterns in different tissues contribute to the processes of drug absorption, distribution and elimination, which determine local drug concentrations at the site of action [9]. ABC transporters, such as ABCB1/MDR1 and ABCC1/MRP1, efflux drugs and metabolites out of the cells; the activity of these transporters is implicated in determining drug bioavailability and development of multidrug resistance [10]. ABC transporters can interact with a diverse range of substrates, including chemotherapeutic agents (e.g. irinotecan, doxorubicin, methotrexate), cardioactive drugs (e.g. digoxin), lipid‐lowering drugs (statins), endogenous compounds (e.g. cholesterol) and antiviral agents (e.g. saquinavir, adefovir, tenofovir) [11]. Depending on the abundance and tissue distribution of transporters, similar drug levels in the systemic circulation may not reflect local drug kinetics in a given tissue in different individuals. Physiologically based pharmacokinetic (PBPK) models that incorporate quantitative transporter data can be used to address both pharmacokinetic sources of variability and pharmacodynamic variations in response to therapy [12, 13].

Despite recent advances in proteomic techniques [14], quantification of large and low‐abundance transmembrane proteins, such as ABC transporters, remains challenging. Mass spectrometry (MS)‐based proteomics offers significant advantages over traditional antibody‐based methods, mainly selectivity and sensitivity. In addition, the ability to determine the levels of several transporters simultaneously by liquid chromatography–mass spectrometry (LC‐MS) affords the possibility to uncover global changes in expression, as well as correlations between different transporters and with other relevant proteins [15]. The use of mass spectrometry to quantify human transporters has been reviewed recently [10]. Targeted proteomic techniques are used to quantify a specific set of proteins selected before the experiment (with the requirement for suitable target‐specific standards), while global proteomics can be used to quantify very large numbers of proteins without prior information [14, 16]. In the last 10 years, targeted proteomic approaches have been extensively used to measure several ABC transporters in different human tissues, including the liver [17, 18, 19, 20], brain [21, 22, 23], lungs [24], kidneys [19, 25, 26, 27] and intestine [17, 28, 29, 30]. These measurements have been incorporated into various translational pharmacology applications, such as the prediction of drug–drug interactions [31] and investigation of the effects of disease [21, 32], ethnicity [33] and ontogeny [34].

Global proteomic approaches have started to attract more interest, owing to the huge improvement in resolution and sensitivity of state‐of‐the‐art mass spectrometry over the last decade, which enabled wider proteome coverage to be achieved [14]. To illustrate this point, recent studies reported quantification of over 2000 proteins, including seven ABC transporters, in 23 human liver samples [15], and more than 3000 proteins in microvessels from 22 human brains, which included 6 ABC transporters [21]. In global proteomics, a survey scan is performed to detect peptide ions, followed by selection of the most intense peaks for fragmentation. Spectra are used for peptide sequence identification by database searching, and subsequently, peptides are assigned to proteins. Signal intensity from global proteomic analysis is used for quantification either against exogenous protein standards [15] or by the ‘total protein approach’ (TPA) [35]. The TPA method is a simple quantification approach for large‐scale proteome characterization without the need for standards or complex normalization steps.

In recent projects, we have generated various global proteomic data sets across multiple human tissues, presenting a unique opportunity to interrogate these data sets for relevant biological questions. The aim of this study was to compare the abundance and tissue distribution of ABC transporters in five human tissues (adult liver, paediatric liver, kidney, intestine, brain and skin). These data sets were generated in the same laboratory using similar techniques and therefore should allow direct comparison and meaningful biological conclusions to be drawn.

## Materials and methods

### Study ethics and sample collection

A total of nine sets of samples making 200 samples in total were processed during the period 2016–2019. Samples were procured opportunistically as surgical surplus or postmortem, and therefore, a power calculation was not used for study design. The liver sample sets are designated Adult Liver Sets 1 and 2, Paediatric Liver Sets 1 and 2 and Biliary Atresia (BA) Livers for clarity.

Ethics approval and the collection of human tissue samples were obtained for brain (*n* = 22), intestine (*n* = 16) and kidney (*n* = 20) as previously described [21, 30, 36]. Adult liver microsomes (Adult Liver Set 1, *n* = 27) were obtained from Pfizer (Groton, CT, USA) as previously described [15]. Human adult livers (Adult Liver Set 2, *n* = 39), removed from histologically normal tissue adjacent to tumours, were obtained from the Manchester Royal Infirmary, Manchester University NHS Foundation Trust, UK, with ethical approval for use in research from North West Research Ethics Committee, UK (14/NW/1260, 19/NW/0644 [37]. Skin tissues (*n* = 6) were obtained from individuals undergoing routine abdominoplasty surgery by the Teaching Hospital of the University of Bradford; ethical approval for use in research was given by the Independent Ethics Committee (36‐DRMBPY‐06‐001) [38]. In addition, two living skin equivalent (LSE) models were supplied by Labskin UK Ltd (York, UK); these were delivered after 14 days of development in transport culture medium. LSE is treated as a single skin sample in this report, but are described in more detail elsewhere [38]. Paediatric Liver Set 1 was made up of samples (*n* = 8) provided by Invitron (Monmouth, UK) and samples (*n* = 12) obtained as microsomes from XenoTech (Lenexa, KS, USA). BA Livers (*n* = 25) were obtained from Ethical Tissue University of Bradford Biobank (study covered under the Ethical Tissue University of Bradford Biobank generic ethics approval 07/H1306/98 and 17/YH/0086). Control (Paediatric Liver Set 2, *n* = 24), nonliver disease‐associated tissue samples were obtained from Erasmus University Medical Centre (Leiden, The Netherlands), covered under the University of Manchester ethics approval 2018‐0892‐5651.

Tissue samples were collected through either surgical procedures (skin, intestine, kidney, Adult Liver Set 2, BA Livers) or postmortem (brain, Adult Liver Set 1, Paediatric Liver Sets 1 and 2). Paediatric samples were categorized based on the European Medicines Agency (EMA) recommendations: fetal (*n* = 5), neonates (0–1 month, *n* = 15), infants (1–23 months, *n* = 8), children (2–11 years, *n* = 12), adolescents (11–17 years, *n* = 3), except that one liver sample from Paediatric Liver Set 1 was from an 18 years old and fitted much more naturally with the adolescents than with the adults. Data for this sample were assigned to the appropriate age group in data analysis. Two of the samples (one infant and one child) showed very few peptides (0 and 1 from ABC transporters) in LC‐tandem mass spectrometry (MS/MS) analysis (see below) and were excluded later in the protocol.

The microsomes and tissue samples were stored at −80 °C until use. Associated demographic information, medical history and other clinical information are listed in the Supplementary Tables of the respective published studies and summarized in Table S16 of the present report.

### Data classification

In total, the data sets were from 200 samples from five organs. The five normal human tissues comprised 66 adult liver tissues, 44 paediatric liver tissues (two were excluded from later analysis), 12 brain tissues, 20 kidney tissues, 16 intestine tissues, six skin tissues plus one Labskin model. The diseased samples included 25 paediatric livers associated with BA and 10 brain samples from donors with Alzheimer’s disease or dementia with Lewy bodies.

### Proteomic sample preparation

Detailed information about tissue and membrane protein preparation of adult livers, paediatric livers, intestine and brain was described previously [15, 21, 30, 36, 37, 39]. Liver and kidney samples were processed to microsomes, whereas brain and intestine tissues were processed to microvessels and mucosal fractions, respectively. For skin, S9 fraction (postmitochondrial fraction) was prepared [38]. Briefly, the subcutaneous layers were removed before skin samples were further processed. Samples were placed in radioimmunoprecipitation assay buffer and were homogenized using a digital handheld homogenizer TT‐30K (Cambio Ltd., Cambridge, UK). After this procedure, the homogenates were centrifuged and supernatants were collected in a clean tube. The supernatants were used for proteomics without any further enrichment.

The proteomic sample preparation for all samples was performed using filter‐aided sample preparation (FASP) as previously described [21]. Digestion protocols were based upon endopeptidase Lys‐C, followed by trypsin. Crude membrane proteins were spiked with stable isotope‐labelled MetCAT and/or TransCAT [40] in the case of Adult Liver Set 2, intestine, kidney and brain microvessel samples (these are examples of Quantification conCATemer (QconCATs), peptide standards concatenated to form a single artificial protein) or with a mixture of exogenous protein standards at known concentrations in the case of paediatric livers, Adult Liver Set 1 [15] and skin samples [38]. The external standards used in the latter set consisted of BSA, horse myoglobin and yeast aldehyde dehydrogenase (ADH).

### Mass spectrometry data acquisition and analysis

In this study, we aimed to analyse a range of in‐house available tissue‐related proteomic data sets obtained in human tissues. Data were based on mass spectrometry (MS) protein measurements in various tissue samples. The raw data contained output from a combination of sources, including enriched and crude fractions. Two types of LC‐MS/MS platforms, Orbitrap Elite and Q Exactive HF mass spectrometers (Thermo Fischer Scientific, Bremen, Germany), were used as previously described [15, 21, 36, 37, 39].

All data analysis was performed using maxquant version 1.6.7.0 (Max Planck Institute, Martinsried, Germany) [41]. The database used for MaxQuant search was a customized database, which included human UniProtKB proteome (UP000005640), containing 71 790 sequences (Oct 2019), supplemented with forward and reverse sequences, in addition to five in‐house QconCAT sequences (designed for the analysis of human enzymes and transporters) and four sequences of standard proteins (ALBU_BOVIN, MYG_HORSE, CYC_BOVIN and ADH1_YEAST).

Mass tolerance of 5 p.p.m. was used for precursor ions and 0.5 Dalton for fragment ions. The search included cysteine carbamidomethylation as a fixed modification. Peptide methionine oxidation was set as a variable modification. Up to one missed cleavage was allowed for trypsin/P digestion. The peptide false discovery rate was set as 1%, and peptides with a minimum of seven amino acid length were considered. For QconCAT peptide identification, ^13^C_6_ Lys and ^13^C_6_ Arg were set as labels. Evidence data files were used for global proteomic data analysis. Identification of proteins was based on unique and ‘razor’ peptides as described previously [21, 37]. Individual MS raw files were analysed without the ‘match between runs’ option. Proteins matching the reverse database were filtered out.

After initial processing, the MaxQuant data were stripped of protein identifiers and rebuilt. A global razor was constructed by assigning any nonunique peptides to as many proteins for which unique peptides could be detected. This was done on a data set by data set basis, so that if a unique peptide was detected in one or more samples in any data set, nonunique peptides could be used as a basis for quantification in the other samples. This approach was deemed superior to treating each sample independently because ABC transporters are close to the limit of detection by LC‐MS/MS and in some cases have considerable sequence homology. The absence of unique peptides is, however, noted in Table S2 (summary). The razor involves first ranking proteins by number of detected peptides and then by order in ‘Human Proteome CAPKR10’. This bespoke database (21 234 sequences) was constructed from the reduced (one sequence, one protein) UniProt [42] Human Protein fasta file, available at https://www.uniprot.org/proteomes/UP000005640 with additional proteins (from the full UniProt database used by MaxQuant) added when they were detected in our samples. Finally, the database was organized so that intact proteins were favoured over fragments and cDNA‐derived proteins, and long primary sequences were favoured over short sequences. The steps taken to complete the assignments and quantification are described in detail in a series of tutorial video files, available on request.

This database contains a small number of single nucleotide polymorphic variants of the ABC transporters ABCB3 and ABCC6.

### Razor

A razor was constructed in a specific way for these proteins. Firstly, we used a global razor, rather than a specific sample razor. Thus, if there were unique peptides from a particular protein in one or more samples, that protein was deemed to be present in all samples in the set, even if the unique peptides fell below the limit of quantification. The limit of quantification was defined based on reproducibility of replicate analyses, as per standard practice in global proteomic analyses [14]. Secondly, there is very high homology between, for example, ABCB1, ABCB4 and ABCB11, which account for most of the shared peptides, and also between ABCA1 and ABCA2. By default, intensities attributable to common peptides from two or more proteins are assigned to each protein equally; however, this approach is inappropriate where one of the contributing proteins is much more abundant than another. Thus, for relevant ABC transporter peptides, the intensities of unique peptides were totalled across all samples in the data set, and intensity due to any shared peptides assigned according to the ratio of unique peptide intensities, as shown in Table S3.

Because the processing algorithms were complex and manual, two operators processed each data set independently.

### Protein quantification across tissues

We have previously applied several approaches for quantification of transporters and enzymes in these samples. For consistency, we here performed (re)quantification for all samples by applying the TPA [35, 43], modified as described above. The TPA method calculates the ratio of individual protein MS signal intensity to total proteome MS signal intensity.

### Data analysis and annotation

Abundance data analysis was carried out using Microsoft Excel 2016, graphpad prism 8.3.0 (San Diego, CA, USA) and r 3.6.0 (collaborative software https://www.r‐project.org/contributors.html). Expression data were presented as mean and SD as a measure of variability across donors. Principal component analysis (PCA) was applied to quantitative data for 32 ABC transporters across five tissues to assess differences in expression patterns. Ontogeny was assessed by amalgamation of data from paediatric and adult liver batches based on EMA age classification into fetal (before birth), neonatal (0–1 month), infant (1–23 months), child (2–11 years), adolescent (11–17 years) and adult (> 18 years) groups. Data from different batches were binned together, and mean values (with SE) were used to plot ontogeny trajectories; only transporters detected in all age groups were assessed for ontogeny. The effect of BA on paediatric expression of ABC transporters was assessed using an unpaired two‐tailed *t*‐test relative to age group‐matched controls (neonates and infants). A probability cut‐off of 0.05 was considered for statistical significance.

The mass spectrometry proteomic data have been deposited to the ProteomeXchange Consortium *via* the PRIDE [44] partner repository with the data set identifiers PXD020910 (Adult Liver Set 1), PXD021025 (Adult Liver Set 2), PXD020844 (Paediatric Liver Set 1), PXD020939 (Paediatric Liver Set 2), PXD020974 (BA Livers), PXD021018 (brain microvessels), PXD020996 (kidney), PXD020987 (small intestine) and PXD020742 (skin).

## Results

### Overview of sample sets

Samples (*n* = 200) were assigned to nine sets (Table 1) depending upon tissue, chronology and experimenter. Sample preparations were based on (liver and kidney) microsomes, (brain) microvessels [45], (intestine) mucosal fractions and (skin) S9 fractions. All preparation methods had the effect of concentrating the membrane fractions that contain the ABC transporters, but none was a targeted isolation of these proteins. Samples were prepared using consistent methodology, based on the FASP protocol [46, 47], with digestion using endopeptidase Lys‐C followed by trypsin.

**Table 1:** Average number of peptides detected in each set of samples (×1000).

Samples	Adult Liver Set 1	Adult Liver Set 2	Paediatric Liver Set 1	Paediatric Liver Set 2	Biliary Atresia Livers	Kidney	Intestine	Brain	Skin
Average number of peptides	15.2	6.7	12	8.4	10.2	16.25	24.6	6.5	10.1
Range	14–17	5–9*	7–15	3–14	5–13	6–23	18–31	3–9	6–15

* A single outlier sample (BA29) from Adult Livers Set 2 gave only 1400 peptides.

In the present study, we used a modified TPA method [35] for label‐free quantification, which does not require standards. Most of the samples, however, contained either QconCATs [40] or standard proteins used for global analysis by the Top 3 approach so that complementary measurements could be made using the same samples [15]. The addition of small quantities of standard proteins is unlikely to influence any aspect of the protocol, except that QconCATs (which are labelled with ^13^C_6_‐arginine and ^13^C_6_‐lysine) may contribute a small amount of residual unlabelled material which must be accounted for, as described previously [48]. Targeted approaches were used for the following sets: Adult Liver Set 2 [37], brain [21], intestine [39] and kidney (Al‐Majdoub *et al*., in preparation). The overlap between targets quantified in previous reports [21] and the current study was not extensive and included only three ABC transporters (ABCA2, ABCB1 and ABCG2), and the values were within 1.7‐fold.

The 200 raw files, most with replicates, were processed using maxquant [41]. The liver samples were grouped according to data set rather than according to demographic, so that the data from groups of identically processed samples run on the same instrument on the same day could be used for internal validation.

### Numbers of identified peptides

The overall number of peptides detected per sample varied considerably with the type of sample and technology (Table 1). Adult Liver Set 1 and kidney microsomes yielded similar numbers of peptides. Adult Liver Set 2 was processed on an older, less sensitive mass spectrometer than Set 1. This impacted on low‐abundance proteins resulting in relatively poor detection of ABC transporters. Table S4 indicates the total number of peptides detected in each sample, which averaged 15 200 peptides for Adult Liver Set 1 and 6700 peptides for Adult Liver Set 2 (Table 1). Available tissue mass for paediatric liver samples was generally smaller and gave weaker LC‐MS/MS results than the corresponding adult samples. Microvessels from brain samples were highly purified and despite long LC gradients (3 h against 90 min for liver microsomes) gave relatively small numbers of proteins and peptides. Conversely, the very large numbers of peptides identified in the intestinal samples probably reflect the use of crude membrane fractions. Skin S9 fractions were similarly more heterogeneous, reflective of the more challenging sample processing due to large amounts of fat remaining attached after surgery yielding these samples.

### Quantification of ABC transporters and other proteins

In the modified TPA used here, the signal due to peptides attributable to more than one ABC transporter was divided according to the ratio of intensity of unique peptides, as described above. This nuance is important because of the high homology between ABC transporter proteins. Inclusion of the total intensity for each of the shared signals for each protein will result in overestimation, whereas excluding nonunique peptides will result in underestimation. The TPA is well suited to quantifying proteins with few unique peptides and is a good indicator of the relative amounts of different proteins. It is less well suited to absolute quantification, tending to overestimate by factors of around 2–3 (El‐Khateeb *et al*., unpublished). This is, in part, because all individual protein intensities are compared with the total detected protein; the protein content that falls below the limit of detection (BLD) is not considered. In the current report, we focus on the relative abundances of proteins in these samples, but the amounts of ABC transporters in pmol·mg^−1^ are given in Tables S5‐S14.

**Fig. 1:**
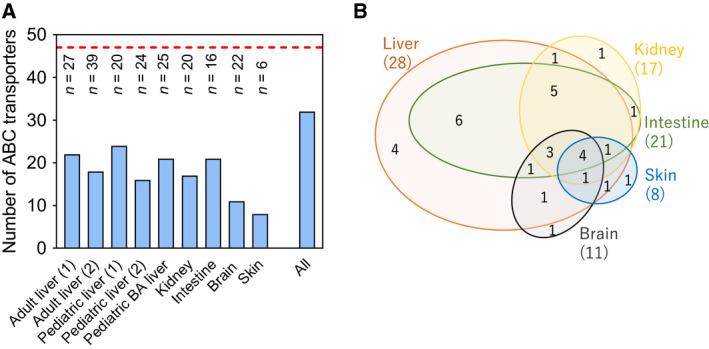
Qualitative analysis of the expression of ABC transporters in five human tissues. (A) The number of ABC transporters expressed in adult liver (two sets), paediatric liver (three sets), kidney, intestine, brain and skin samples. The number of samples is indicated above the bars. The number of ABC transporters ranged from 8 to 24 in each set, with a total of 32 transporters. (B) Venn diagram of the overlap of expressed ABC transporters between liver, kidney, intestine, brain and skin. The numbers in parentheses are the numbers of transporters expressed in each organ. The number of ABC transporters in liver is the cumulative number from five (adult and paediatric) liver sets.

**Fig. 2:**
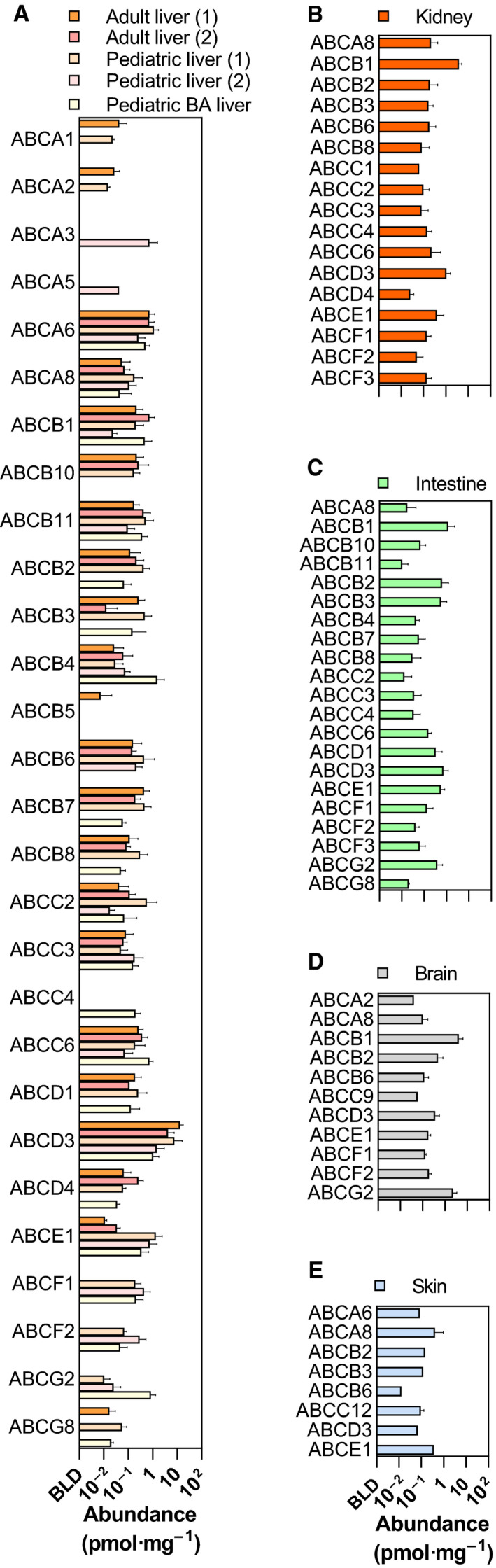
Distribution and abundance of human ABC transporters. (A) Adult and paediatric liver, (B) kidney, (C) intestine, (D) brain and (E) skin. BA refers to biliary atresia samples from the paediatric liver set. Samples with transporters BLD were not considered in quantitative analysis. Units of abundance are pmol of ABC transporter per mg total protein in samples. Error bars represent SD.

Table 2 lists the frequencies of detection of ABC transporters for each of the nine sets of samples. Of the 48 ABC transporters encoded by the human genome (ABCA11P is a pseudogene), 32 could be detected and quantified in at least some samples. Seventeen (ABCA4, ABCA7, ABCA9, ABCA10, ABCA11, ABCA12, ABCA13, ABCB9, ABCC5, ABCC7, ABCC8, ABCC10, ABCC11, ABCD2, ABCG1, ABCG4 and ABCG5) fell BLD in these samples by global proteomic techniques (Fig. 1A). In addition, ABCC12, found only in skin samples, was actually close to the limit of detection and not found if alternative processing parameters were applied [38]. The Venn diagram in Fig. 1B shows that four transporters, ABCA8, ABCB2, ABCD3 and ABCE1, were detected across all five tissues. Expression levels and tissue distribution of quantified ABC transporters are shown in Fig. 2.

**Table 2:** Detection frequency of ABC transporters in each tissue set. Values represent numbers of samples in each set where the transporters were detected. ABC transporters are at low abundance, and the absence of any transporter in any sample means only that it falls BLD. For example, ABCG5 and ABCG8 form an obligatory dimer, but ABCG5 is never detected. This indicates that its peptides are less easily detected than those of ABCG8.

Transporter	Adult Liver Set 1 (*n* = 27)	Adult Liver Set 2 (*n* = 39)	Paediatric Liver Set 1 (*n* = 20 age 2 w −18 y)	Paediatric Liver Set 2 (*n* = 24, fetal −7 y)	Biliary Atresia Livers (*n* = 25, 2 w‐5 m)	Brain (*n* = 22)	Intestine (*n* = 16)	Kidney (*n* = 20)	Skin (*n* = 7)
ABCA1	All	None	4	None	None	None	None	None	None
ABCA2	21	None	4	None	None	2	None	None	None
ABCA3	None	None	None	2	None	None	None	None	None
ABCA5	None	None	None	1	None	None	None	None	None
ABCA6	All	38	All	9	24	None	None	None	1
ABCA8	18	15	13	5	21	7	7	All	2
ABCB1 (P‐gp)	All	23	All	12	23	All	All	All	None
ABCB2 (TAP1)	24	23	All	None	8	13	All	16	1
ABCB3 (TAP2)	All	6	19	None	14	None	All	10	1
ABCB4	All	33	18	20	17	None	All	None	None
ABCB5	4	None	None	None	None	None	None	None	None
ABCB6	23	6	17	10	None	4	None	17	1
ABCB7	All	24	14	None	10	None	All	None	None
ABCB8	All	11	14	None	6	None	All	15	None
ABCB10	All	15	12	None	None	None	All	None	None
ABCB11	All	All	All	17	All	None	10	None	None
ABCC1	None	None	None	None	None	None	None	1	None
ABCC2	24	All	12	2	8	None	4	18	None
ABCC3	25	33	9	4	24	None	14	5	None
ABCC4	None	None	None	None	20	None	All	All	None
ABCC6	All	All	All	12	24	None	All	18	None
ABCC9	None	None	None	None	None	1	None	None	None
ABCC12	None	None	None	None	None	None	None	None	3
ABCD1	26	1	6	None	3	None	None	All	None
ABCD3	All	38	All	17	24	8	All	All	1
ABCD4	24	None	3	None	6	None	3	None	None
ABCE1	7	17	11	13	22	6	All	15	2
ABCF1	None	3	11	5	8	3	All	19	None
ABCF2	None	None	4	3	18	18	4	7	None
ABCF3	None	None	None	None	None	None	9	15	None
ABCG2	None	None	4	4	24	All	All	None	None
ABCG8	10	None	3	None	4	None	2	None	None
A4, A7, A9, A10, A11, A12, A13, B9, C5, C7, C8, D2, G1, G4, G5	not detected								

### Polymorphic ABC transporters

Polymorphisms were detected for two ABC transporters. ABCC6 has two variants listed in the UniProt database, designated as A0A0G2JM3_HUMAN and MRP6_HUMAN. The peptide GAL**V**CCLDQAR from A0A0G2JM3_HUMAN was found in several liver samples, but the corresponding GAL**M**CCLDQAR from MRP6_HUMAN was not. Conversely, LVTFLCLEEVDPG**V**VDSSSSGSAAGK, assigned to MRP6_HUMAN, appeared in one liver sample, but the corresponding LVTFLCLEEVDPG**A**VDSSSSGSAAGK from A0A0G2JM3_HUMAN was not found in these data sets. A composite entry ABCC6_HUMAN containing the observed peptides was added to our database to accommodate these variations. As for transporter associated with antigen processing (TAP)2 (ABCB3), both NNI**A**YGLQSCEDDK and NNI**T**YGLQSCEDDK peptides were detected, and data were analysed using the same approach. The two peptides were not detected in all samples. The wild‐type NNIAYGLQSCEDDK was detected in 16 of the 20 samples in paediatric set 1, and in a few of the other samples. The corresponding NNITYGLQSCEDDK was detected in four samples from the same set, 3 of which were certainly heterozygous (the wild‐type peptide was also detected). Where both peptides were detected, intensities were similar (within a factor of 2). Although a large difference in response cannot be ruled out, it would not be expected for two such similar peptides. Fig. 2 reflects quantitative data for the two transporters (ABCB3 and ABCC6) across the five tissues using the outlined strategy.

### ABC Transporters in brain, skin, intestine and liver

The brain microvessel samples showed relatively high amounts of ABCB1 (MDR1) compared with other tissues with no evidence of ABCB4 or ABCB11. ABCG2 (BCRP) was the next most highly represented ABC transporter. The high expression of these efflux transporters protects the brain from xenobiotics, including drugs. There is considerable interest in the regulation of these transporters in Alzheimer's disease. Reports about ABCG2 in Alzheimer's disease are conflicting, with different groups suggesting that it is unchanged or upregulated [49, 50]. Similar amounts of ABCG2 were detected in our diseased and control brain microvessel samples, albeit with a small number of samples (5 and 12, respectively) [21]. There is more compelling evidence that ABCB1 (MDR1) shows reduced activity in Alzheimer's disease [51, 52, 53, 54, 55] and that inhibition of ubiquitination can alleviate symptoms of the disease. We did not find reduced ABCB1 in diseased brain microvessel samples compared with control; however, mass spectrometry does not necessarily distinguish between ubiquitinated and nonubiquitinated proteins. Ubiquitinated protein would be expected to have zero or reduced function. Interestingly, ABCD3 is detected only in nondiseased brain microvessel samples, indicating that it may be downregulated in neurodegenerative disease. The detection of TAP1 (ABCB2), in the absence of TAP2 (ABCB3), is observed only in the brain microvessel samples. This was unexpected, as TAP1 and TAP2 normally form a heterodimer. A previous study reported the functional significance of TAP1 (but not TAP2) expression in tumour cells [56]. Functional data on mouse tumour cells support an independent role for Tap1 in cytotoxic T lymphocyte‐mediated lysis and reduction in brain metastasis. Overall, the brain microvessel samples gave rise to good preliminary data, indicating those ABC transporters that are relatively abundant, such as ABCG2, and worthy of further investment (such as targeted methodology on a much larger sample set). See Table S6 for full results.

The aim of the original skin study was to quantify xenobiotic‐metabolizing enzymes, and any conclusions about ABC transporters must be treated cautiously. ABCA8 was robustly identified, but the TPA used here failed to identify and quantify ABCB11, which was quantified by more conventional (standard‐based) data processing (Couto *et al*., accepted for publication in Drug Metabolism and Disposition). See Table S8 for full results.

We previously published a targeted analysis to quantify intestinal protein abundance of drug‐metabolizing enzymes and transporters using the same samples [30]. It is gratifying that the quantification obtained using global analysis in the current study broadly agrees for ABCB1 and ABCG2. ABCC2 (MRP2) peptides were generally below the limit of quantification in this study, whereas quantification was achieved using the targeted approach, which is, as indicated earlier, generally more sensitive. The advantage of a label‐free approach is that targets do not need to be defined in advance, and thus, proteins such as TAP1 and TAP2 (ABCB2 and ABCB3) may be quantified. 21 ABC transporter proteins were quantifiable in at least some intestinal samples, and ABCB1 [P‐glycoprotein (P‐gp)], ABCB2 (TAP1), ABCB3 (TAP2) and ABCB4, the mitochondrial transporters ABCB7, ABCB8 and ABCB10, ABCC4, ABCC6 (MRP4 and MRP6), ABCD1, ABCD3, ABCE1, ABCF1 and ABCG2 (BCRP) were quantifiable in all 16 samples. See Table S5 for full results.

Three ABC transporters were detected in all 20 kidney samples. These were ABCB1 (P‐gp), which was overwhelmingly the most abundant, ABCA8 and ABCD3. ABCA8 is an anion pump and transporter of lipids. A preliminary report suggested that the mycotoxin ochratoxin A is a substrate for ABCA8 and that transport through the kidney causes nephrotoxicity [57]; the role of ABCA8 is to decrease the extent of toxicity by effluxing the toxin. ABCC1 and ABCC4 transporters are also important in the kidney and efflux many endogenous molecules and drug conjugates (glucuronides in particular). See Table S7 for full results.

In Adult Liver Set 1, ABCD3 was the most abundant ABC transporter by more than an order of magnitude, followed by ABCA6. ABCB7, ABCB10, ABCC6, ABCB1 and ABCB3 (TAP2) were also significantly represented. Adult Liver Set 2 gave much weaker signals because a different mass spectrometer was used for sample analysis; nevertheless, ABCD3>ABCA6 were clearly the most abundant ABC transporters, followed by ABCB1>ABCB11>ABCC6>ABCB7, ABCB10, ABCE1 and ABCB2. Paediatric Liver Set 1 included samples from children aged 3–18 years, mostly above 2 years. In these paediatric samples, ABCD3 was also the most abundant, followed by ABCA6>ABCE1>ABCB2, ABCB3 and ABCB11. Paediatric Liver Set 2 represented particular experimental difficulties due to the very small mass of sample available, and legal requirement for processing to be carried out in two different countries. Two samples from Paediatric Liver Set 2 (one infant and one child) were excluded from consideration in this study because peptides assigned to ABC transporters could not be detected. On average, ABCD3 remained the most abundant ABC transporter followed by ABCE1>ABCA6. The samples from biliary atresia patients were by far the most homogeneous in terms of demographics. All patients were a few weeks to a few months old, and all had the same disease. Four ABC transporters appeared to be significantly more abundant than the others: ABCD3, ABCB4>ABCG2 and ABCC6. (See Table S9‐S10 for control paediatric livers, Table S11 for BA livers and Tables S12‐13 for adult livers.)

### Global tissue‐related expression patterns

**Fig. 3:**
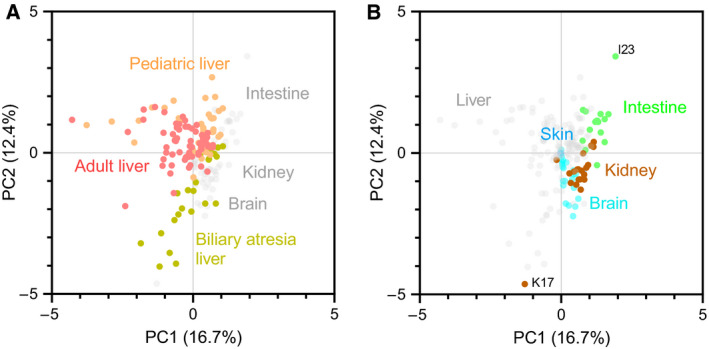
Multivariate analysis of the expression of ABC transporters in five tissues. (A) PCA of expression in adult livers, paediatric livers and diseased paediatric livers (BA); paediatric livers and adult livers showed similar expression profiles and diseased livers clustered away from controls. (B) PCA of expression in kidney, intestine, brain and skin samples; data points describing expression in kidney, intestine and brain clustered away from liver, with clear distinction of the three organs. Outliers from kidney and intestine clusters are labelled. PC1 and PC2 represent the first and second principal components with the largest proportion of explained variance as indicated by the percentages in parentheses.

Assessing expression in the nine sets independently was informative, but the strength of the present study is the opportunity to compare patterns across tissues. We therefore assessed expression data of ABC transporters in all sets using multivariate analysis (Fig. 3). There were two overarching observations. Paediatric livers and adult livers overlapped, showing variable but similar expression profiles across 4 data sets (Adult Liver Sets 1 and 2 and Paediatric Liver Sets 1 and 2); the diseased livers (BA Livers), however, clustered away from controls (Fig. 3A). The second trend was related to the other organs (kidney, intestine, brain and skin), which showed expression patterns different from liver (clustering away from all five liver sets), and a clear distinction (with small overlap) between the sets of the three organs (Fig. 3B). The five sets of liver samples provided the most comprehensive coverage.

### Ontogeny of ABC transporters in the liver

**Fig. 4:**
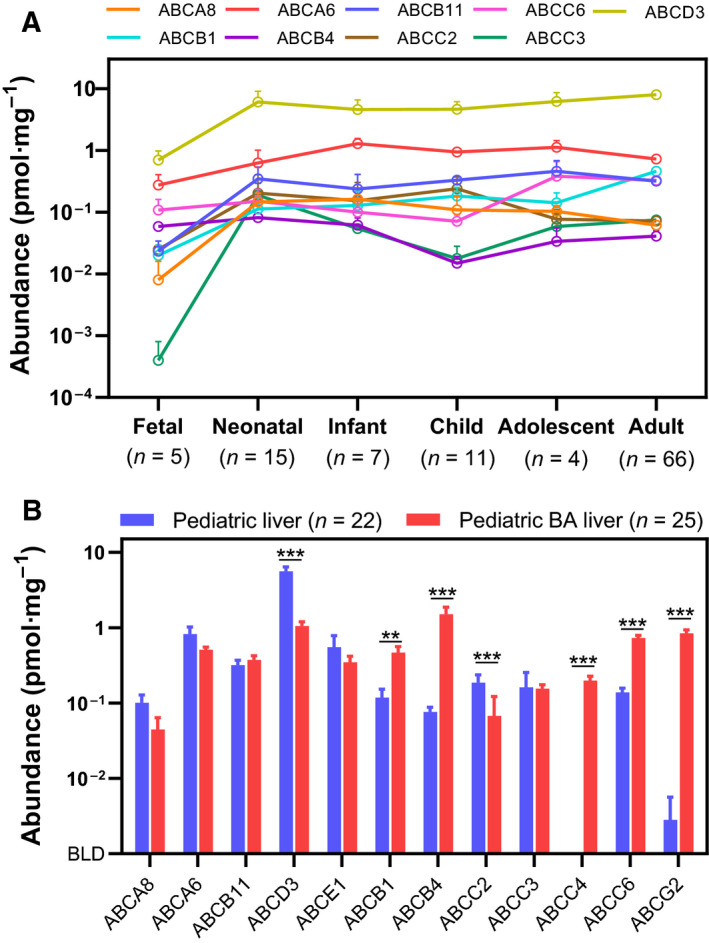
Ontogeny and effect of biliary atresia on expression of hepatic ABC transporters. (A) Ontogeny trajectories show that transporters reached maturity in the first few months of life. (B) BA livers showed different transporter expression patterns compared with paediatric livers from neonates and infants, with higher expression levels of ABCB1, ABCC4, ABCC6 and ABCG2. Error bars represent SE.

Ontogeny was considered in Table S14 and Fig. 4A, where samples were divided into fetal, neonate (up to 1 month), infant (1 month–2 years), children (2–12 years), adolescents (12–18 years) and adults.

Small differences in sample preparation can obscure ontogeny because differences in sample preparation affect the transporter abundance data when expressed (as is usual) in pmol·mg^−1^ total protein. Attempts were made to compare the transporter intensity values with marker proteins such as ATP1A1, but the effect of sample set was not removed. The mean values (and SE) of expression of key ABC transporters in different age groups are shown in Fig. 4A (with the data from BA Livers excluded). In most cases shown (ABCA6, ABCA8, ABCB1, ABCB11, ABCC2, ABCC3 and ABCD3), there is a sharp rise in abundance between fetal and neonatal samples, followed by levelling off or more gradual increase. ABCB4 and ABCC6 show imperceptible increase in very early life. The increase in both protein abundance and mRNA expression of ABCB1 with age has been reported previously [34, 58, 59, 60, 61, 62, 63]. Prasad *et al*. [34] showed stable expression of ABCB11 from childhood to adulthood and our results are consistent with these, but show a marked rise between fetal and neonate samples. In our experiments, ABCB4 appeared approximately constant across the different age groups, although data obtained by immunohistochemistry [58, 63] suggest that this transporter also increases in abundance with age.

### Biliary atresia

Biliary atresia is a rare liver disease of young children in which bile ducts are severely narrowed or blocked. Although the disease almost always leads to liver transplant, the Kasai surgical procedure [64] delays the need for this transplant from early childhood to, typically, late adolescence. In this procedure, the damaged bile ducts are replaced by small sections of intestinal tissue and liver samples are obtained during this procedure. Outcomes appear to be best when the procedure is performed as early as possible (in the first few weeks of life); consequently, all 25 samples originate from children of similar age (2 weeks to 5 months). Fig. 4B shows comparison of ABC transporter levels between BA and control paediatric samples (age group‐matched: neonates and infants), showing downregulation of ABCD3, ABCC2 and ABCA8 compared with control levels. Notably, ABCG2 and ABCC4 are almost absent in controls but abundant in biliary atresia; ABCB1 and ABCB4 are both significantly upregulated to the point that ABCB4 is the most abundant transporter in BA Livers, overtaking ABCD3. This is the first report of ABC transporter abundance in biliary atresia. Expression of the plasma membrane marker ATP1A1 (used as negative control) in the control paediatric and disease groups was within 1.7‐fold, indicating that the observed differences are likely due to impact of disease rather than technical error.

## Discussion

Active transport by ABC transporters contributes to many processes that define drug disposition. Perturbations in transporter expression or functional activity are readily noted from the measurement of drug concentration in plasma. For example, inhibition of intestinal BCRP (ABCG2) has been implicated in the drug–drug interaction between rosuvastatin (substrate) and fostamatinib (inhibitor) [65]. For some drugs, ABC transporters have critical roles in drug elimination from the systemic circulation, *via* the liver (e.g. MRP2/ABCC2) or kidney (e.g. P‐gp/ABCB1). Finally, ABC transporters may contribute to selective and asymmetric drug distribution in tissues, which would not be apparent from only measuring drug concentration in plasma.

As such, the role of ABC transporters in defining local concentration at pharmacological target sites in the body (as opposed to the systemic circulation) has largely been ignored. The exception perhaps has been the role of ABC transporters at the blood–brain barrier where their significant role, particularly for psychoactive drugs, has been documented [66]. Elements such as genetic variations or environmentally driven regulation of transporter abundance and functionality can affect the disparity between the systemic and local drug concentrations. Our increasing knowledge of the ABC transporters, combined with PBPK modelling and simulation of local drug concentrations, is now allowing us to make these distinctions [67]. However, many data gaps remain, particularly relating to potential differences in the abundance and activity of transporters between subgroups of patients. Proteomic abundance data for ABC transporters are therefore needed to realise the full potential of pharmacokinetic models. In this report, we present quantified values of ABC transporters in five different tissues, totalling 200 human samples, by LC‐MS/MS‐based proteomics. A cross‐tissue comparison was not the primary purpose of these experiments, and the results are therefore hypothesis‐generating rather than hypothesis‐testing.

Compared to enzymes, ABC transporters are poorly abundant and were close to the limit of detection in all these experiments, and therefore, coverage was mixed. Where samples were of reasonable size and the most recent mass spectrometers available were used, coverage was good, as shown with the sample sets termed Adult Liver Set 1, Paediatric Liver Set 1, intestine, kidney and (perhaps surprisingly) BA Livers. Consistent with hypothesis generation, global proteomic analysis was performed, but improvements in sensitivity are expected where targeted methods are used in hypothesis testing [21, 30]. Key findings are summarized below.

### Polymorphisms in ABCB3 and ABCC6 are observed

Typically, 3000–30 000 peptides per sample were detected in total; from these, 32 of the 48 ABC proteins were securely detected and quantified in at least some of the samples. The global approach allowed the detection of two polymorphisms – in TAP2 (ABCB3) and MRP6 (ABCC6). These polymorphisms were detectable because both appear in the UniProt human proteome database. This is a rather unusual situation – normally this UniProt database defines a wild‐type protein and variants are not detected. However, it points to the possibility of creating bespoke databases (fasta files) for proteomics, in which important polymorphisms are explicitly included. In the case of TAP2, both versions of the peptide NNIAYGLQSCEDDK/ NNITYGLQSCEDDK appear. Tang *et al*. [68] reported using genotyping analysis that the homozygous (A565T) variant is particularly prevalent in Zimbabweans and Zambians (18.3%), Brazilians (10.1%), but Caucasians and Rwandans almost lack this variant with frequencies of (0.7% and 0%), respectively. The heterozygous form is found in Caucasians (1.3%) but is much more common among Zambians (35%) and Brazilians (20.3%). Of the four samples in which A565T was detected, three (one male Caucasian, one male Asian and one unknown) were certainly heterozygous. In the fourth sample (also male Caucasian), the wild‐type peptide was not observed, but it is likely that this was a technical, not a biological, absence. To our knowledge, this is the first example of polymorphisms of ABC transporters being detected by LC‐MS/MS. There is, however, increasing interest in using this technique to monitor polymorphisms relevant to drug metabolism and disposition, and so far, cytochrome P450 2B6, uridine 5'‐diphospho‐glucuronosyltransferase 2B15 and carboxylesterase 1 have been explicitly quantified as more than one allele [69, 70, 71].

### ABCD3 is the most abundant liver ABC transporter

Across all liver samples, ABCD3 was the most abundant ABC transporter and was also detected significantly in the kidney. The ABCD series are peroxisomal transporters involved in the import of branched‐chain and very long fatty acids [72]. ABCD3 also plays a role in bile acid transport into peroxisomes, a key step in bile acid homeostasis [73]. ABCD transporters regulate cellular response to oxidative stress and control inflammatory response by regulating peroxisomal β‐oxidation of fatty acids [74]. Both ABCD1 and ABCD3 have low tissue specificity, and they are downregulated in renal cancer [74, 75]. ABCD3 is also downregulated in colorectal cancer [75]. Downregulation leads to lipid accumulation in tissue which promotes carcinogenesis, indicating a protective role for peroxisomal transporters [72]. The two sets of adult liver samples had different origins: Adult Liver Set 1 was opportunistically derived from sudden death victims (including cardiovascular disease, road traffic accidents, gunshot wounds), whereas Adult Liver Set 2 was surgical samples from cancer patients (normal tissue adjacent to cancer). The mean abundances for ABCD3 are consistent with this reported downregulation in cancer (Liver Set 1: 13.2 ± 0.9 pmol·mg^−1^ microsomal protein; Liver Set 2: 4.1 ± 0.5 pmol·mg^−1^ microsomal protein).

### TAP1 is expressed more highly than TAP2 in brain microvessels

There is strong evidence that TAP1 (ABCB2) is expressed in human brain microvessels at a much higher level than its partner, TAP2. TAP2 was detected neither in this study nor when an alternative processing package (Progenesis) was used [21]. TAP2 was detected and quantified at similar levels to TAP1 in liver, kidney, intestine and even skin. An independent role for TAP1 in the brain has been suggested [56] and that is consistent with these observations. The obligatory dimer ABCG5/ABCG8 behaves differently. ABCG8 was detected at low level in some samples, but ABCG5 was not detected in these experiments. We attribute this consistent pattern to technical differences between the two proteins – ABCG8 giving rise to more readily detected peptides than its partner.

### Ontogeny of ABCB1, ABCB4 and ABCB11

Global LC‐MS/MS techniques allow thousands of proteins to be detected and quantified simultaneously, with very high selectivity, hence the potential for hypothesis generation. The selectivity allows for ABCB1, ABCB4 and ABCB11 to be clearly distinguished on the basis of their unique peptides, and this, in turn, allows us to postulate that ABCB1 and ABCB11 sharply increase in abundance between fetal and neonatal samples; our data suggest that ABCB4 is the dominant fetal form.

### The liver ABC transporter profile of biliary atresia patients differs from normal paediatric liver

The transporter profile for BA Livers is particularly exciting with significant upregulation of four transporters (ABCB1, ABCB4, ABCC4 and ABCG2) compared with controls and downregulation of three others (ABCA8, ABCC2 and ABCD3). Biliary atresia, extrahepatic ductopenia or progressive obliterative cholangiopathy is a disease of childhood with unknown genetic origin. Here, bile ducts are very narrow or even blocked. One might speculate that the upregulation of ABCB1, ABCB4, ABCC4 and ABCG2 is due to their function in detoxification; especially, ABCC4 is often referred to as a ‘backup system in liver cholestasis’. However, these results should be interpreted cautiously, as the ‘control’ samples were all taken postmortem; they are control in the sense of having died from something other than liver disease. In this case, however, the disease samples show marked variation from adult, also, providing additional confidence in these findings.

### Study limitations

It is important to exercise extreme caution in the interpretation of global LC‐MS/MS proteomic data. While we can be confident of the successful identification of ABC transporters by LC‐MS/MS, failure to detect a transporter does not mean that it is not there. The abundance of a transporter may be BLD, which depends upon the characteristics of the proteins, and their corresponding peptides, in sample preparation, liquid chromatography and mass spectrometer [76]. Even the data processing package can influence the detection of low‐abundance proteins [77, 78]; when we processed the Adult Liver Set 1 with an alternative software package, only 65% of the quantifiable peptides were common (A.‐M. Vasilogianni, E. El‐Khateeb, S. Alrubia, Z.M. Al‐Majdoub, N. Couto, B. Achour, A. Rostami‐Hodjegan, & J. Barber, unpublished).

The high homology of some ABC transporters presents additional problems. In a small number of cases, we observed only nonunique peptides corresponding to a particular transporter in a sample. Where unique peptides corresponding to that protein were detected in similar samples, we have postulated that the protein is present – one example is ABCA2 in adult livers, for which unique peptides were detected in some samples, but in other samples, only peptides shared with ABCA1 were detected. By contrast, we were unable to detect ABCC7 in intestinal samples where mRNA measurements and immunoblotting might lead us to expect it [79, 80]. mRNA correlates poorly with protein concentrations, and immunoblotting is especially susceptible to interference from closely related proteins, such as ABCB1 and ABCC8 [18, 81, 82, 83, 84].

A further complication for proteomics in the drug metabolism and disposition arena is the inheritance of the unit pmol·mg^−1^ total protein from the drug metabolism community. This is potentially problematic as different results will inevitably be obtained with different sample preparations. The idea of referencing to grams of tissue is gaining traction [14] to reconcile differences in tissue processing and overcome problems inherent to normalization against independently regulated proteins [20, 85]. This normalization approach is not immediately suitable for historical samples (where yield and loss scalars to tissue content are not available), which are often stored as membrane fractions.

The liver and kidney samples were processed to extract enriched microsomal fractions in order to enable quantification of enzymes and transporters. To avoid additional protein loss, no further purification was attempted to extract plasma membrane fractions.

Targeted LC‐MS/MS is more suitable than global LC‐MS/MS for hypothesis testing, combining higher sensitivity and more rigorous quantification but at the expense of multiplexing. Targeted experiments to confirm the ABC transporter profile in BA with a view to modelling drug efflux from the liver in this disease are especially important.

In conclusion, LC‐MS/MS brings unique insights into the distribution and abundances of ABC transporters. The sensitivity and selectivity of the technique and corresponding abundance of data must not, however, lead to overconfidence. There are difficulties in sample procurement and sample preparation, and MS/MS data are inherently poorly reproducible. When used with care, LC‐MS/MS‐generated proteomic data can powerfully complement the results of other studies and open up new avenues of enquiry.

## Author contributions

ZMA, BA, NC, MH, YE and DS performed the experimental work. ZMA, BA, SA, EE‐K, A‐MV JB analysed the data. ZMA, BA, SN, LS, AR‐H and JB synthesized and interpreted the results. All authors prepared the manuscript.

## Supporting information


**Table S1.** ABC transporters in human tissue.
**Table S2.** Number of distinct (D) peptides (unique peptides and other assigned to a single protein in this dataset), other (O, not distinct) peptides and the range of distinct peptides per sample (DS) for each transporter in each tissue.
**Table S3.** The division of common peptide intensities among ABC transporters.
**Table S4.** Total numbers of peptides (thousands) in each sample.
**Table S5.** Expression levels of quantifiable ABC transporters in intestine samples.
**Table S6.** Expression levels of quantifiable ABC transporters in brain samples.
**Table S7.** ABC transporter abundance pmol·mg^−1^ protein in individual kidney samples.
**Table S8.** ABC transporter abundance pmol·mg^−1^ protein in individual skin samples.
**Table S9.** ABC transporter abundance pmol·mg^−1^ protein in individual Paediatric Livers Set 1 samples.
**Table S10.** ABC transporter abundance pmol·mg^−1^ protein in individual Paediatric Livers Set 2 samples.
**Table S11.** ABC transporter abundance pmol·mg^−1^ protein in Biliary Atresia Livers.
**Table S12.** ABC transporter abundance pmol·mg^−1^ protein in Adult Livers Set 1 samples.
**Table S13.** ABC transporter abundance pmol·mg^−1^ protein in Adult Livers Set 2 samples.
**Table S14.** Ontogeny of ABC transporters in the human liver. Abundances are given as mean of non‐zero values ± standard error in pmol per mg total protein.
**Table S15.** The number of males and females in each sample set.
**Table S16.** Demographic and clinical details of all datasets.
