# Supplementary material for: Mass spectrometry‐based abundance atlas of ABC transporters in human liver, gut, kidney, brain and skin
Source: FEBS Lett. 2020 Dec 3;594(23):4134–50. doi: 10.1002/1873-3468.13982 (PMC7756589; doi:10.1002/1873-3468.13982)
Supplement: Supplementary file 1 — Table S1. ABC transporters in human tissue. Table S2. Number of distinct (D) peptides (unique peptides and other assigned to a single protein in this dataset), other (O, not distinct) peptides and the range of distinct peptides per sample (DS) for each transporter in each tissue. Table S3. The division of common peptide intensities among ABC transporters. Table S4. Total numbers of peptides (thousands) in each sample. Table S5. Expression levels of quantifiable ABC transporters in intestine samples. Table S6. Expression levels of quantifiable ABC transporters in brain samples. Table S7. ABC transporter abundance pmol·mg−1 protein in individual kidney samples. Table S8. ABC transporter abundance pmol·mg−1 protein in individual skin samples. Table S9. ABC transporter abundance pmol·mg−1 protein in individual Paediatric Livers Set 1 samples. Table S10. ABC transporter abundance pmol·mg−1 protein in individual Paediatric Livers Set 2 samples. Table S11. ABC transporter abundance pmol·mg−1 protein in Biliary Atresia Livers. Table S12. ABC transporter abundance pmol·mg−1 protein in Adult Livers Set 1 samples. Table S13. ABC transporter abundance pmol·mg−1 protein in Adult Livers Set 2 samples. Table S14. Ontogeny of ABC transporters in the human liver. Abundances are given as mean of non‐zero values ± standard error in pmol per mg total protein. Table S15. The number of males and females in each sample set. Table S16. Demographic and clinical details of all datasets. [file FEB2-594-4134-s001.docx]

**Supplementary Information**

Mass Spectrometry-Based Abundance Atlas of ABC Transporters in Human Liver, Gut, Kidney, Brain, and Skin

Zubida M. Al-Majdoub, Brahim Achour, Narciso Couto, Martyn Howard, Yasmine Elmorsi, Daniel Scotcher, Sarah Alrubia, Eman Elkhateeb, Areti-Maria Vasilogianni, Noura Alohali, ***Sibylle Neuhoff***, Lutz Schmidt, Amin Rostami-Hodjegan, Jill Barber

**Supplementary Information Content**

**Supplementary Table 1.** ABC transporters in human tissue.

**Supplementary Table 2.** Number of distinct (D) peptides (unique peptides and other assigned to a single protein in this dataset), other (O, not distinct) peptides and the range of distinct peptides per sample (DS) for each transporter in each tissue.

## Supplementary Table 3. The division of common peptide intensities among ABC transporters.

## Supplementary Table 4. Total numbers of peptides (thousands) in each sample.

**Supplementary Table 5.** Expression levels of quantifiable ABC transporters in intestine samples.

**Supplementary Table 6.** Expression levels of quantifiable ABC transporters in brain samples.

**Supplementary Table 7.** ABC transporter abundance pmol per milligram protein in individual kidney samples

**Supplementary Table 8.** ABC transporter abundance pmol per milligram protein in individual skin samples

**Supplementary Table 9**: ABC transporter abundance pmol per milligram protein in individual paediatric livers set 1 samples.

**Supplementary Table 10**: ABC transporter abundance pmol per milligram protein in individual paediatric livers set 2 samples.

**Supplementary Table 11**: ABC transporter abundance pmol mg^-1^ protein in biliary atresia samples

**Supplementary Table 12**: ABC transporter abundance pmol mg^-1^ protein in adult liver samples set 1

**Supplementary Table 13**: ABC transporter abundance pmol mg^-1^ protein in adult liver samples set 2

**Supplementary Table 14**: Ontogeny of ABC transporters in the human liver. Abundances are given as mean of non-zero values ± standard error in pmol per mg total protein.

**Supplementary Table 15**: The number of males and females in each sample set.

**Supplementary Table 16**: Demographic and clinical details of all datasets.

# **Supplementary Table 1.** ABC transporters in human tissue

| **Gene name** | **Other names** | **Notes** | **Tissue specificity (Liver, Intestine, Kidney, Skin, Brain)** | **References** |
| --- | --- | --- | --- | --- |
| **ABCA1** | ABC1, CERP | Cholesterol and high density lipoproteins (HDL) transporter, gene polymorphisms associated with the risks of diabetes mellitus, dyslipidemia and Tangier disease. | All | [1,2] |
| **ABCA2** | ABC2, KIAA1062 | Involved in drug resistance. |  |  |
| **ABCA3** | ABC3 | Surfactant lipid transporter, mutations in ABCA3 cause severe lung disease. Upregulation of ABCA3 in cerebrospinal Fluid (CSF) is associated with vestibular schwannoma. | All | [3,4] |
| **ABCA4** | ABCR | Photoreceptor-specific transporter associated with Stargardt disease, a form of macular degeneration. | Eye specific | [5] |
| **ABCA5** | KIAA1888 | Cholesterol & lipid transporter regulates amyloid-β protein precursor (AβPP) processing associated with Alzheimer’s disease (AD) pathology. | All | [6] |
| **ABCA6** |  | ABCA6 plays a role in macrophage lipid homeostasis. Up-regulated during monocyte differentiation into macrophages. Down-regulated by cholesterol loading of macrophages. | All | [7] |
| **ABCA7** | ABCA-SSN | ABCA7 plays a role in lipid homeostasis, transportation, and macrophage-mediated phagocytosis. Expression pattern reflects a role in lipid homeostasis in immune system cells. Common variants are associated with Alzheimer’s disease. | All (strongly expressed in bone marrow and lymph nodes) | [8] |
| **ABCA8** | KIAA0822 | ABCA8 facilitates cholesterol efflux and modulates high density lipoprotein cholesterol (HDLc). Mutations are associated with lower plasma HDLc. High expression is associated with Multiple system atrophy as brain disease. | All | [9,10] |
| **ABCA9** |  | May play a role in monocyte differentiation and lipidhomeostasis. High expression was reported in ovarian cancer. | All (mainly ovary and fats) | [11,12] |
| **ABCA10** | ABCAA | ABCA10 may play a role in macrophage lipid homeostasis. | All (mainly ovary and fats) | [13] |
| **ABCA11P** | ABCA11 | Lacks ABC transporter domains. Defined as a pseudogene by Hugo gene nomenclature committee (HGNC). Plays a role in ATPase activity. | All | [14,15] |
| **ABCA12** | ABC12, ABCAC | Probable transporter involved in lipid homeostasis. Mutations in its genes are associated with Ichthyosis, congenital, autosomal recessive 4A. | Mainly expressed in the stomach, skin, placenta, testis, kidney and foetal brain | [16] |
| **ABCA13** | ABCAD | Plays role in lipid transport and neutrophil degranulation. Rare variants are associated with neurodegenerative diseases. | All | [17] |
| **ABCB1** | MDR1, P-gp | Multi-drug resistance, mutations are associated with drug resistance in cancer. | All except skin | [18] |
| **ABCB2** | TAP1 | Peptide half transporter (forms a dimer with TAP2) with a role in antigen presentation during immune response. | All | [19] |
| **ABCB3** | TAP2 | Peptide half transporter (forms a dimer with TAP1) with a role in antigen presentation during immune response. | All | [19] |
| **ABCB4** | MDR3 | Phosphatidylcholine transport, dysfunction is associated with intrahepatic cholestasis and low-phospholipid-associated cholelithiasis syndrome. | Liver specific | [20] |
| **ABCB5** | ABCB5 P-gp | ABCB5 alleles is associated with drug induced CNS toxicity. | Eye specific | [21] |
| **ABCB6** | MTABC3, PRP, UMAT | Mitochondrial iron transporter. Impaired polymorph results in porphyria. | All | [22,23] |
| **ABCB7** | ABC7 | Mitochondrial half transporter, mutation of ABCB7 causes abnormal iron, fatty acid metabolism and sideroblastic anemia. | All | [24,25] |
| **ABCB8** | M-ABC1 | Mitochondrial half transporter, able to form complexes with other proteins, protecting against oxidative stress, also associates with melanoma drug resistance. | All | [26,27] |
| **ABCB9** | KIAA1520 | Half transporter involved in peptide transport (forms homodimer) with a role in antigen presentation during immune response. | All | [19] |
| **ABCB10** | ABCBA, M-ABC2 | Mitochondrial transporter, play a role in peptide export and active protection from increased oxidative stress. ABCB10 deletion is associated with Huntington's disease. | All | [28,29] |
| **ABCB11** | ABCBB, BSEP | Bile salt transporter involved in cholestatic liver disorders. Mutations in ABCB11 cause progressive familial intrahepatic cholestasis type 2 (PFIC2). | Liver specific | [30] |
| **ABCC1** | MRP1 | Drug resistance and protective role from toxins. | All | [31] |
| **ABCC2** | MRP2 | Drug resistance. Dysfunction is associated with diseases such as Dubin-Johnson syndrome. | All except skin | [32] |
| **ABCC3** | MRP3, CMOAT2, MLP2 | It is involved in the biliary and intestinal excretion of organic anions. It expresses in different cancer types, such as human hepatocellular carcinoma, lung cancer and gallbladder cancer. | All (mainly expressed in liver and gallbladder) | [33–35] |
| **ABCC4** | MRP4 | Organic anion pump associated with drug resistance and possibly cellular detoxification. It is involved in prostaglandin secretion. Its expression is altered in various cancer types. | All (strongly expressed in prostate) | [36–39] |
| **ABCC5** | MRP5 | Drug resistance. It is a multi-specific organic anion pump enabling the transport of nucleotide analogues. | All | [40] |
| **ABCC6** | MRP6 | ABCC6 actively transport organic anions into subcellular organelles. Mutations in ABCC6 is associated with an autosomal recessive heritable disorder called Pseudoxanthoma elasticum. ABCC6 homozygous variant is critically important to ABCC6 function. | Kidney and liver (very low expression in other tissues) | [41,42] |
| **ABCC7** | CFTR | ABCC7 involves in transmembrane active transport and fluid homeostasis. Mutation is associated with cystic fibrosis. | All (mainly Pancreas and colon, except brain) | [43,44] |
| **ABCC8** | SUR1, HRINS | ABCC8 mutation causes congenital hyperinsulinism as it regulates insulin release by encoding ATP-sensitive potassium channels. | All (mainly brain, pancreas, and pituitary gland) | [45] |
| **ABCC9** | SUR2 | Participate in regulation and activation of cardiac and smooth muscle ATP-sensitive potassium channels. Influence sleep duration. | All (mainly heart) | [46,47] |
| **ABCC10** | MRP7, SIMRP7 | Resistance capabilities for taxanes anticancer drugs. A lipophilic anion transporter participate in cellular detoxification. | All | [48,49] |
| **ABCC11** | MRP8 | Abnormal expression decreases cellular cAMP and cGMP and resistant to purine and pyrimidine nucleotide analogues. It is a lipophilic anions uptake transporter. | All (except kidney, brain, spleen and colon) | [50,51] |
| **ABCC12** | MRP9 | Both ABCC11 and ABCC12 are linked to paroxysmal kinesigenic choreoathetosis disorder. | Mainly testis and brain | [52] |
| **ABCD1** | ALD | Transport free very-long-chain fatty acids and their CoA-esters. Its lack causes X-linked adrenoleukodystrophy genetic disorder. | All | [53] |
| **ABCD2** | ALD1 | ABCD2 mutations observed in neurodegenerative diseases such as adrenoleukodystrophy and Zellweger syndrome. | Brain and heart | [54] |
| **ABCD3** | PMP70 | Transporting hydrophilic fatty acids. Genetic mutations lead to hepatosplenomegaly and severe liver diseases including hepatic fibrosis. | All | [55,56] |
| **ABCD4** | PXMP1L | Involved in releasing lysosomal vitamin B12 (cobalamin). Mutations lead to vitamin B12 deficiency. | All | [57,58] |
| **ABCE1** | RLI, RNASEL1, RNASELI | A host factor for viral replication. Inhibits RNase L endoribonuclease activity. Regulates of mRNA turnover. Antagonizes the anti-viral effect of the interferon-regulated 2-5A/RNase L pathway. | All | [59] |
| **ABCF1** | ABC50 | Plays a role in regulating innate immunity. Up-regulated in autoimmune pancreatitis and rheumatoid arthritis. | All | [60] |
| **ABCF2** | HUSSY-18 | Mutations have been involved in cancer progression. Plays a role in Cisplatin resistance in ovarian adenocarcinomas. | All | [61] |
| **ABCF3** |  | Exhibits anti-flavivirus response. Lacks transmembrane domains and is probably not involved in transport. Displays antiviral effect against flaviviruses in the presence of OAS1B. | All | [62] |
| **ABCG1** | ABC8, WHT1 | Cholesterol and phospholipids efflux, regulate cellular lipid distribution. | All | [63] |
| **ABCG2** | BCRP, MXR | Efflux of toxins, resistance to drugs, involved in urate metabolic imbalances such as gout and hyperuricemia. | All except skin | [64] |
| **ABCG4** | WHITE2 | Contributes in the efflux of desmosterol and amyloid-β peptide (Aβ) at the blood brain barrier. | Brain | [65] |
| **ABCG5** | WHITE3 | ABCG5 and ABCG8 form an obligate heterodimer that mediates Mg2+- and ATP-dependent sterol transport across the cell membrane. These two transports contribute to intestinal cholesterol homeostasis. | Gut & Liver (mRNA evidence) | [66] |
| **ABCG8** |  | ABCG8 allele is associated with gallstone disease. | All | [67] |

## Supplementary Table 2. Number of distinct (D) peptides (unique peptides and other assigned to a single protein in this dataset), other (O, not distinct) peptides and the range of distinct peptides per sample (DS) for each transporter in each tissue.

| **Name** | **Adult Livers Set 1** | | | **Adult Livers Set 2** | | | **Paediatric Livers Set 1** | | | **Paediatric Livers Set 2** | | | **Biliary atresia livers** | | |
| --- | --- | --- | --- | --- | --- | --- | --- | --- | --- | --- | --- | --- | --- | --- | --- |
|  | D | O | DS | D | O | DS | D | O | DS | D | O | DS | D | O | DS |
| **ABCA1 (ABC1,CERP)** | 15 | 2 | 0-10 |  |  |  | 1 | 0 | 0-1 |  |  |  |  |  |  |
| **ABCA2 (ABC2, KIAA1062)** | 1 | 2 | 0-1 |  |  |  |  |  |  |  |  |  |  |  |  |
| **ABCA3 (ABC3)** |  |  |  |  |  |  |  |  |  | 10 | 0 | 0-10 |  |  |  |
| **ABCA5 (KIAA1888)** |  |  |  |  |  |  |  |  |  | 1 | 0 | 0-1 |  |  |  |
| **ABCA6** | 45 | 0 | 3-27 | 28 | 0 | 0-14 | 30 | 1 | 6-21 | 8 | 0 | 0-7 | 17 | 1 | 0-10 |
| **ABCA7 (ABCA-SSN)** | 0 | 1 |  |  |  |  |  |  |  |  |  |  |  |  |  |
| **ABCA8 (KIAA0822)** | 9 | 0 | 0-6 | 9 | 0 | 0-4 | 16 | 2 | 0-11 | 5 | 0 | 0-4 | 9 | 1 | 0-7 |
| **ABCA9** | 0 | 1 | 0 |  |  |  |  |  |  | 0 | 1 |  |  |  |  |
| **ABCA13 (ABCAD)** | 0 | 0 | 0 |  |  |  |  |  |  | 0 | 1 |  |  |  |  |
| **ABCB1 (MDR1, P-gp)** | 16 | 8 | 1-9 | 3 | 6 | 0-3 | 13 | 5 | 0-9 | 2 | 1 | 0-2 | 4 | 3 | 0-3 |
| **ABCB2 (TAP1)** | 10 | 0 | 0-8 | 8 | 0 | 0-7 | 7 | 0 | 1-6 |  |  |  | 3 | 0 | 0-2 |
| **ABCB3 (TAP2)** | 10 | 1 | 1-6 | 3 | 1 | 0-1 | 9 | 1 | 0-7 |  |  |  | 5 | 0 | 0-4 |
| **ABCB4 (MDR3)** | 7 | 8 | 0-5 | 9 | 6 | 0-5 | 6 | 5 | 0-4 | 2 | 1 | 0-2 | 2 | 3 | 0-2 |
| **ABCB5 (ABCB5 P-gp)** | 1 | 1 | 0-1 |  |  |  |  |  |  |  |  |  |  |  |  |
| **ABCB6 (MTABC3, PRP, UMAT)** | 9 | 1 | 0-6 | 3 | 0 | 0-1 | 6 | 0 | 0-6 | 3 | 0 | 0-2 |  |  |  |
| **ABCB7 (ABC7)** | 17 | 0 | 2-10 | 8 | 0 | 0-4 | 9 | 0 | 0-7 |  |  |  | 3 | 0 | 0-2 |
| **ABCB8 (M-ABC1)** | 13 | 1 | 0-9 | 5 | 1 | 0-1 | 0 |  |  |  |  |  | 2 | 0 | 0-2 |
| **ABCB10 (ABCBA, M-ABC2)** | 12 | 1 | 1-8 | 5 | 1 | 0-2 | 6 | 1 | 0-4 |  |  |  |  |  |  |
| **ABCB11 (ABCBB, BSEP)** | 23 | 2 | 0-13 | 22 | 2 | 1-9 | 21 | 2 | 0-15 | 7 | 0 | 0-4 | 7 | 1 | 1-4 |
| **ABCC2 (MRP2)** | 16 | 0 | 0-13 | 13 | 0 | 0-7 | 20 | 0 | 0-15 | 2 | 0 | 0-1 | 6 | 0 | 0-2 |
| **ABCC3 (MRP3, CMOAT2, MLP2)** | 19 | 0 | 0-12 | 8 | 0 | 0-3 | 4 | 0 | 0-3 | 6 | 0 | 0-5 | 3 | 1 | 0-3 |
| **ABCC4 (MRP4)** |  |  |  |  |  |  |  |  |  |  |  |  | 1 | 1 | 0-1 |
| **ABCC6 (MRP6)** | 25 | 0 | 4-14 | 20 | 0 | 2-11 | 15 | 0 | 1-10 | 5 | 0 | 0-5 | 7 | 1 | 0-2 |
| **ABCC12 (MRP9)** | 0 | 1 | 0 |  |  |  |  |  |  |  |  |  |  |  |  |
| **ABCD1 (ALD)** | 14 | 0 | 0-7 | 2 | 0 | 0-1 | 7 | 0 | 0-6 |  |  |  | 3 | 0 | 0-3 |
| **ABCD2 (ALDL1)** | 0 | 1 | 0 |  |  |  |  |  |  |  |  |  |  |  |  |
| **ABCD3 (PMP70)** | 31 | 0 | 17-25 | 32 | 0 | 0-17 | 29 | 0 | 4-21 | 9 | 0 | 0-6 | 16 | 0 | 0-8 |
| **ABCD4 (PXMP1L)** | 5 | 0 | 0-4 |  |  |  | 4 | 0 | 0-2 |  |  |  | 2 | 0 | 0-1 |
| **ABCE1 (RLI, RNASEL1, RNASELI)** | 2 | 0 | 0-1 | 7 | 0 | 0-3 | 10 | 0 | 0-8 | 10 | 0 | 0-7 | 14 | 0 | 0-8 |
| **ABCF1 (ABC50)** |  |  |  | 3 | 0 | 0-1 | 11 | 0 | 0-5 | 8 | 0 | 0-7 | 4 | 0 | 0-3 |
| **ABCF2 (HUSSY-18)** |  |  |  |  |  |  | 2 | 0 | 0-1 | 6 | 0 | 0-5 | 2 | 0 | 0-2 |
| **ABCG2 (BCRP,MXR)** |  |  |  |  |  |  | 1 | 0 | 0-1 | 1 | 0 | 0-1 | 2 | 0 | 0-2 |
| **ABCG8** | 2 | 0 | 0-2 |  |  |  | 2 | 0 | 0-1 |  |  |  | 1 | 0 | 0-1 |

D= distinct, O= other, DS= distinct per sample

| **Name** | **Brain** | | | **Intestine** | | | **Kidney** | | | **Skin** | | |
| --- | --- | --- | --- | --- | --- | --- | --- | --- | --- | --- | --- | --- |
|  | D | O | DS | D | O | DS | D | O | DS | D | O | DS |
| **ABCA2 (ABC2,KIAA1062)** | 1 | 0 | 0-1 |  |  |  |  |  |  |  |  |  |
| **ABCA6** |  |  |  |  |  |  |  |  |  | 4 | 0 | 0-4 |
| **ABCA8 (KIAA0822)** | 2 | 0 | 0-2 | 8 | 0 | 0-8 | 22 | 0 | 2-12 | 16 | 0 | 0-16 |
| **ABCB1 (MDR1, P-gp)** | 20 | 0 | 5-15 | 38 | 8 | 5-31 | 42 | 9 | 10-34 |  |  |  |
| **ABCB2 (TAP1)** | 5 | 0 | 0-3 | 14 | 1 | 4-14 | 10 | 0 | 0-6 | 3 | 0 | 0-3 |
| **ABCB3 (TAP2)** |  |  |  | 16 | 1 | 2-15 | 13 | 0 | 0-6 | 2 | 0 | 0-2 |
| **ABCB4 (MDR3)** |  |  |  | 2 | 8 | 1-2 |  |  |  |  |  |  |
| **ABCB6 (MTABC3, PRP, UMAT)** | 1 | 0 | 0-1 |  |  |  | 8 | 0 | 0-4 | 1 | 0 | 0-1 |
| **ABCB7 (ABC7)** |  |  |  | 9 | 0 | 1-5 |  |  |  |  |  |  |
| **ABCB8 (M-ABC1)** |  |  |  | 5 | 0 | 0-3 | 7 | 1 | 0-4 |  |  |  |
| **ABCB10 (ABCBA, M-ABC2)** |  |  |  | 7 | 1 | 0-5 |  |  |  |  |  |  |
| **ABCB11 (ABCBB, BSEP)** |  |  |  | 1 | 2 | 0-2 |  |  |  |  |  |  |
| **ABCC1 (MRP1)** |  |  |  |  |  |  | 2 | 1 | 0-3 |  |  |  |
| **ABCC2 (MRP2)** |  |  |  | 5 | 0 | 1-4 | 14 | 0 | 0-8 |  |  |  |
| **ABCC3 (MRP3, CMOAT2, MLP2)** |  |  |  | 11 | 0 | 0-8 | 4 | 1 | 0-3 |  |  |  |
| **ABCC4 (MRP4)** |  |  |  | 2 | 0 | 1-2 | 17 | 1 | 2-13 |  |  |  |
| **ABCC6 (MRP6)** |  |  |  | 2 | 1 | 2-2 | 17 | 0 | 0-11 |  |  |  |
| **ABCC9 (SUR2)** | 1 | 0 | 0-1 |  |  |  |  |  |  |  |  |  |
| **ABCC12 (MRP9)** |  |  |  |  |  |  |  |  |  | 1 | 0 | 0-1 |
| **ABCD1 (ALD)** |  |  |  | 17 | 0 | 2-16 |  |  |  |  |  |  |
| **ABCD3 (PMP70)** | 3 | 0 | 0-3 | 15 | 0 | 2-10 | 18 | 0 | 3-12 | 2 | 0 | 0-2 |
| **ABCD4 (PXMP1L)** |  |  |  |  |  |  | 3 | 0 | 0-1 |  |  |  |
| **ABCE1 (RLI, RNASEL1, RNASELI)** | 1 | 0 | 0-1 | 17 | 0 | 4-13 | 17 | 0 | 0-11 | 2 | 0 | 0-1 |
| **ABCF1 (ABC50)** | 1 | 0 | 0-1 | 13 | 0 | 1-11 | 11 | 0 | 0-9 |  |  |  |
| **ABCF2 (HUSSY-18)** | 1 | 0 | 0-1 | 2 | 0 | 0-2 | 5 | 0 | 0-4 |  |  |  |
| **ABCF3** |  |  |  | 6 | 0 | 0-4 | 9 | 0 | 0-5 |  |  |  |
| **ABCG2 (BCRP,MXR)** |  |  |  | 7 | 0 | 1-5 |  |  |  |  |  |  |
| **ABCG8** |  |  |  | 2 | 0 | 0-2 |  |  |  |  |  |  |

**Supplementary Table 3.** The division of common peptide intensities among ABC transporters

|  |  | **Ratios** | | | | | | |
| --- | --- | --- | --- | --- | --- | --- | --- | --- |
| **Peptide** | **Assignments** | **Adult liver 1** | **Adult liver 2** | **Paediatric liver set 1** | **Paediatric liver set 2** | **Biliary atresia liver** | **Kidney** | **Intestine** |
| IAIISHGK | ABCA1/A2 | 0.64:0.36 |  |  |  |  |  |  |
| SVVLTSHMEECEALCTR | ABCA1/A2 | 0.64:0.36 |  |  |  |  |  |  |
| TTIVVAHR | ABCB5/B6 | 0.02:0.98 |  |  |  |  | ABCB6 only |  |
| TVLVIAHR | ABCB8/B10/B3 | 0.20:0.38:0.43 | 0.28:0.69:0.03 | 0.27:0.15:0.60 |  |  | 0.48:0:0.52 | 0.04:0.10:0.83 |
| ILLLDEATSALDTESEK | ABCB11/B1/B4 | 0.55:0.40:0.05 | 0.44:0.53:0.03 | 0.72:0.25:0.03 | 0.49:0.10:0.42 | 0.24:0.26:0.50 | ABCB1 only | 0.002:0.96:0.03 |
| TCIVIAHR | ABCB11/B1/B4 | 0.55:0.40:0.05 | 0.44:0.53:0.03 | 0.72:0.25:0.03 |  |  | ABCB1 only | 0.002:0.96:0.03 |
| VKEHGTHQQLLAQK | ABCB1/B4 | 0.90:0.10 |  |  |  |  |  |  |
| EHGTHQQLLAQK | ABCB1/B4 | 0.90:0.10 |  |  |  |  | ABCB1 only | 0.97:0.03 |
| LSTIQNADLIVVFQNGR | ABCB1/B4 | 0.90:0.10 |  |  |  |  |  |  |
| TTIVIAHR | ABCB1/B4 | 0.90:0.10 |  |  |  |  | ABCB1 only | 0.97:0.03 |
| STVVQLLER | ABCB1/B4 | 0.90:0.10 | 0.94:0.06 | 0.88:0.12 |  | 0.34:0.66 | ABCB1 only | 0.97:0.03 |
| KGQTLALVGSSGCGK | ABCB1/B4 | 0.90:0.10 |  |  |  |  |  | 0.97:0.03 |
| GQTLALVGSSGCGK | ABCB1/B4 |  | 0.94:0.06 | 0.88:0.12 |  |  | ABCB1 only | 0.97:0.03 |
| FDTLVGER | ABCB1/B4 |  | 0.94:0.06 | 0.88:0.12 |  |  | ABCB1 only | 0.97:0.03 |
| FDTLVGERGAQLSGGQK | ABCB1/B4 |  |  |  |  | 0.34:0.66 |  |  |
| VVQEALDK | ABCB1/B4 |  | 0.94:0.06 |  |  |  |  |  |
| MWQAIR | ABCA8/PLPL8 |  |  | 0.96:0.04 |  |  |  |  |
| VLLELEMK | ABCA8/A6 |  |  | 0.10:0.80 |  |  |  |  |
| IVSVER | ABCC6/CAVN2 |  |  |  |  |  |  | 0.61:0.39 |
| GLTFTLRPGEVTALVGPNGSGK | ABCB2/B3 |  |  |  |  |  |  | 0.63:0.37 |
| VFLESK | ABCB6/B8 |  |  |  |  | 0.94:0.06 |  |  |
| VGIVGRTGAGK | ABCC3/C4/C6 |  |  |  |  | 0.15:0.14:0.71 |  |  |
| **Brain and skin samples showed only unique peptides for the ABC transporters** | | | | | | | | |

**Supplementary Table 4.** Total numbers of peptides (thousands) in each sample

| **Adult Liver 1** | 01 | 02 | 06 | 08 | 09 | 11 | 25 | 35 | 38 | 41 | 48 | 71 | 72 | 73 | 74 | 75 | 76 | 77 | 78 | 79 | 80 | 89 | 90 | 91 | 100 | 108 | 117 |  |  |
| --- | --- | --- | --- | --- | --- | --- | --- | --- | --- | --- | --- | --- | --- | --- | --- | --- | --- | --- | --- | --- | --- | --- | --- | --- | --- | --- | --- | --- | --- |
| Peptides (1000s) | 14 | 17 | 16 | 15 | 15 | 15 | 16 | 16 | 15 | 15 | 14 | 14 | 15 | 14 | 16 | 14 | 16 | 14 | 16 | 16 | 15 | 15 | 15 | 15 | 15 | 14 | 17 |  |  |
| **Adult Liver 2ZA** | 01 | 02 | 03 | 04 | 05 | 06 | 07 | 08 | 09 | 10 |  |  |  |  |  |  |  |  |  |  |  |  |  |  |  |  |  |  |  |
| Peptides (1000s) | 9 | 9 | 9 | 9 | 8 | 9 | 9 | 9 | 9 | 9 |  |  |  |  |  |  |  |  |  |  |  |  |  |  |  |  |  |  |  |
| **Adult liver 2BA** | 01 | 02 | 03 | 04 | 05 | 06 | 07 | 08 | 09 | 10 | 11 | 12 | 13 | 14 | 15 | 16 | 17 | 18 | 19 | 20 | 21 | 22 | 23 | 24 | 25 | 26 | 27 | 28 | 29 |
| Peptides (1000s) | 6 | 6 | 6 | 6 | 6 | 6 | 7 | 6 | 6 | 6 | 6 | 6 | 6 | 7 | 6 | 6 | 7 | 6 | 6 | 6 | 6 | 5 | 5 | 6 | 5 | 6 | 6 | 7 | 1 |
| **Paediatric liver 1** | 01 | 02 | 03 | 04 | 05 | 06 | 07 | 08 | 09 | 10 | 11 | 12 | 13 | 14 | 15 | 16 | 17 | 18 | 19 | 20 |  |  |  |  |  |  |  |  |  |
| Peptides (1000s) | 10 | 14 | 12 | 13 | 14 | 13 | 14 | 14 | 12 | 7 | 12 | 13 | 11 | 13 | 15 | 14 | 12 | 10 | 14 | 11 |  |  |  |  |  |  |  |  |  |
| **Paediatric liver 2** | 1 | 2 | 3 | 4 | 5 | 6 | 7 | 8 | 9 | 10 | 11 | 12 | 13 | 14 | 15 | 16 | 17 | 18 | 19 | 20 | 21 | 22 | 23 | 24 |  |  |  |  |  |
| Peptides (1000s) | 12 | 9 | 14 | 10 | 14 | 10 | 10 | 10 | 10 | 7 | 6 | 6 | 4 | 6 | 7 | 5 | 4 | 8 | 3 | 4 | 4 | 6 | 6 | 4 |  |  |  |  |  |
| **Biliary atresia livers** | 1 | 2 | 3 | 4 | 5 | 9 | 10 | 11 | 12 | 14 | 15 | 16 | 17 | 18 | 19 | 20 | 21 | 22 | 23 | 24 | 25 | 26 | 27 | 28 | 29 |  |  |  |  |
| Peptides (1000s) | 12 | 11 | 10 | 7 | 11 | 13 | 5 | 5 | 10 | 8 | 11 | 13 | 9 | 11 | 13 | 12 | 7 | 10 | 9 | 10 | 13 | 13 | 12 | 10 | 13 |  |  |  |  |
| **Kidney** | 11 | 12 | 13 | 14 | 15 | 16 | 17 | 18 | 19 | 20 | 21 | 22 | 23 | 24 | 25 | 26 | 27 | 28 | 29 | 30 |  |  |  |  |  |  |  |  |  |
| Peptides (1000s) | 14 | 19 | 20 | 19 | 17 | 22 | 14 | 23 | 11 | 13 | 23 | 18 | 13 | 14 | 15 | 9 | 14 | 14 | 16 | 15 |  |  |  |  |  |  |  |  |  |
| **Intestine** | 01 | 04 | 05 | 10 | 11 | 12 | 15 | 16 | 18 | 19 | 20 | 21 | 22 | 23 | 26 | 28 |  |  |  |  |  |  |  |  |  |  |  |  |  |
| Peptides (1000s) | 31 | 25 | 24 | 29 | 21 | 23 | 20 | 23 | 19 | 24 | 30 | 29 | 26 | 30 | 18 | 21 |  |  |  |  |  |  |  |  |  |  |  |  |  |
| **Brain** | 01 | 02 | 03 | 04 | 05 | 06 | 07 | 08 | 09 | 10 | 11 | 12 | 13 | 14 | 15 | 16 | 17 | 18 | 19 | 20 | 21 | 22 |  |  |  |  |  |  |  |
| Peptides (1000s) | 7 | 9 | 7 | 6 | 7 | 7 | 6 | 7 | 7 | 7 | 4 | 6 | 6 | 3 | 8 | 5 | 7 | 7 | 7 | 7 | 5 | 7 |  |  |  |  |  |  |  |
| **Skin** | 07 | 52 | 56 | 57 | 58 | 60 | LS |  |  |  |  |  |  |  |  |  |  |  |  |  |  |  |  |  |  |  |  |  |  |
| Peptides (1000s) | 10 | 10 | 6 | 8 | 7 | 15 | 15 |  |  |  |  |  |  |  |  |  |  |  |  |  |  |  |  |  |  |  |  |  |  |

**Supplementary Table 5:** ABC transporter abundance per milligram total protein in individual intestine samples. Individual measurements, means, standard deviation values and %CV in individual values are presented. The number n is the number of intestine samples in which a protein was quantified.

| **Transporter** | **Intestine abundance (pmol mg^-1^)** | | | | | | | | | | | | | | | | | | | |
| --- | --- | --- | --- | --- | --- | --- | --- | --- | --- | --- | --- | --- | --- | --- | --- | --- | --- | --- | --- | --- |
|  | **I01** | **I04** | **I05** | **I10** | **I11** | **I12** | **I15** | **I16** | **I18** | **I19** | **I20** | **I21** | **I22** | **I23** | **I26** | **I28** | **Mean** | **SD** | **%CV** | **n** |
| **ABCA8** | 0.004 |  |  | 0.003 |  | 0.005 |  |  | 0.02 |  | 0.01 | 0.08 |  |  | 0.01 |  | 0.02 | 0.02 | 136 | 7 |
| **ABCB1 (MDR1)** | 0.12 | 0.28 | 2.56 | 1.18 | 1.77 | 0.94 | 1.59 | 0.52 | 1.03 | 4.43 | 2.11 | 0.25 | 0.47 | 0.18 | 0.61 | 1.03 | 1.19 | 1.09 | 91 | 16 |
| **ABCB2 (TAP1)** | 0.36 | 0.41 | 0.61 | 0.62 | 0.25 | 0.40 | 0.21 | 0.87 | 0.54 | 0.38 | 0.54 | 0.30 | 0.70 | 2.48 | 0.18 | 1.41 | 0.64 | 0.56 | 87 | 16 |
| **ABCB3 (TAP2)** | 0.38 | 0.26 | 1.12 | 0.38 | 0.35 | 0.43 | 0.38 | 0.79 | 0.75 | 0.30 | 0.41 | 0.14 | 0.40 | 1.84 | 0.12 | 1.19 | 0.58 | 0.45 | 77 | 16 |
| **ABCB4 (MDR3)** | 0.04 | 0.04 | 0.04 | 0.04 | 0.08 | 0.03 | 0.04 | 0.05 | 0.03 | 0.05 | 0.03 | 0.02 | 0.05 | 0.02 | 0.06 | 0.07 | 0.04 | 0.02 | 38 | 16 |
| **ABCB7 (ABC7)** | 0.07 | 0.01 | 0.05 | 0.05 | 0.02 | 0.06 | 0.06 | 0.09 | 0.04 | 0.07 | 0.05 | 0.02 | 0.24 | 0.06 | 0.01 | 0.01 | 0.06 | 0.05 | 91 | 16 |
| **ABCB8 (M-ABC1)** | 0.02 | 0.003 | 0.02 | 0.04 | 0.04 | 0.04 | 0.01 | 0.01 | 0.01 | 0.01 | 0.00 | 0.02 | 0.07 | 0.02 | 0.17 | 0.01 | 0.03 | 0.04 | 134 | 16 |
| **ABCB10 (M-ABC2)** | 0.08 | 0.01 | 0.10 | 0.11 | 0.06 | 0.02 | 0.05 | 0.15 | 0.06 | 0.03 | 0.07 | 0.01 | 0.18 | 0.10 | 0.04 | 0.03 | 0.07 | 0.05 | 68 | 16 |
| **ABCB11 (BSEP)** |  |  |  |  | 0.02 |  |  |  |  |  | 0.01 |  | 0.01 |  |  |  | 0.01 | 0.005 | 35 | 3 |
| **ABCC2 (MRP2)** | 0.001 |  | 0.04 |  | 0.01 |  |  |  |  |  |  | 0.01 |  |  |  |  | 0.01 | 0.01 | 99 | 4 |
| **ABCC3 (MRP3)** | 0.06 |  | 0.01 | 0.01 | 0.03 | 0.01 | 0.05 | 0.05 | 0.01 | 0.15 | 0.02 |  | 0.05 | 0.03 | 0.01 | 0.00 | 0.04 | 0.04 | 105 | 14 |
| **ABCC4 (MRP4)** | 0.03 | 0.01 | 0.00 | 0.02 | 0.02 | 0.05 | 0.12 | 0.03 | 0.01 | 0.01 | 0.01 | 0.02 | 0.07 | 0.01 | 0.03 | 0.09 | 0.03 | 0.03 | 98 | 16 |
| **ABCC6 (MRP6)** | 0.13 | 0.06 | 0.11 | 0.13 | 0.24 | 0.16 | 0.20 | 0.17 | 0.19 | 0.14 | 0.12 | 0.13 | 0.16 | 0.07 | 0.20 | 0.29 | 0.15 | 0.06 | 37 | 16 |
| **ABCD1 (ALD)** | 0.20 | 0.33 | 0.52 | 0.18 | 0.21 | 0.24 | 0.52 | 0.97 | 0.15 | 0.09 | 0.44 | 0.03 | 1.07 | 0.05 | 0.06 | 0.21 | 0.33 | 0.30 | 91 | 16 |
| **ABCD3 (PMP70)** | 0.41 | 0.20 | 1.62 | 0.57 | 0.91 | 1.18 | 1.09 | 0.87 | 0.43 | 1.22 | 1.30 | 0.12 | 0.73 | 0.08 | 0.30 | 0.60 | 0.73 | 0.45 | 62 | 16 |
| **ABCE1 (RLI)** | 0.83 | 0.60 | 0.41 | 0.64 | 0.36 | 0.35 | 0.41 | 0.53 | 0.49 | 0.41 | 0.78 | 0.67 | 0.89 | 1.20 | 0.17 | 0.25 | 0.56 | 0.26 | 46 | 16 |
| **ABCF1 (ABC50)** | 0.16 | 0.39 | 0.12 | 0.11 | 0.03 | 0.08 | 0.05 | 0.11 | 0.04 | 0.01 | 0.28 | 0.09 | 0.24 | 0.35 | 0.03 | 0.05 | 0.13 | 0.12 | 86 | 16 |
| **ABCF2 (HUSSY-18)** | 0.06 | 0.04 | 0.02 |  |  |  |  |  |  |  |  |  |  | 0.05 |  |  | 0.04 | 0.02 | 39 | 4 |
| **ABCF3** | 0.07 | 0.13 | 0.05 | 0.01 |  |  |  |  |  |  | 0.09 | 0.03 | 0.04 | 0.14 |  | 0.01 | 0.06 | 0.04 | 70 | 9 |
| **ABCG2 (BCRP)** | 0.22 | 0.03 | 1.01 | 0.58 | 0.68 | 0.33 | 0.82 | 0.20 | 0.16 | 0.54 | 0.54 | 0.11 | 0.35 | 0.07 | 0.24 | 0.38 | 0.39 | 0.27 | 70 | 16 |
| **ABCG8** |  |  |  |  |  |  |  | 0.02 |  |  |  |  | 0.02 |  |  |  | 0.02 | 0.001 | 3 | 2 |

**Supplementary Table 6:** ABC transporter abundance per milligram total protein in individual brain samples. Individual measurements, means, standard deviation values and %CV in individual values are presented. The number n is the number of brain samples in which a protein was quantified.

| **Transporter** | **Brain abundance (pmol mg^-1^)** | | | | | | | | | | | | | | | | | | | | | | | | | |
| --- | --- | --- | --- | --- | --- | --- | --- | --- | --- | --- | --- | --- | --- | --- | --- | --- | --- | --- | --- | --- | --- | --- | --- | --- | --- | --- |
|  | **HB**  **01** | **HB**  **02** | **HB**  **03** | **HB**  **04** | **HB**  **05** | **HB**  **06** | **HB**  **07** | **HB**  **08** | **HB**  **09** | **HB**  **10** | **HB**  **11** | **HB**  **12** | **HB**  **13** | **HB**  **14** | **HB**  **15** | **HB**  **16** | **HB**  **17** | **HB**  **18** | **HB**  **19** | **HB**  **20** | **HB**  **21** | **HB**  **22** | **Mean** | **SD** | **%CV** | **n** |
| **ABCA2 (ABC2)** |  |  | 0.04 |  | 0.04 |  |  |  |  |  |  |  |  |  |  |  |  |  |  |  |  |  | 0.04 |  |  | 2 |
| **ABCA8** | 0.03 |  | 0.14 |  |  |  | 0.10 |  |  | 0.05 |  |  |  |  |  |  | 0.23 | 0.08 |  |  | 0.09 |  | 0.10 | 0.06 | 60 | 7 |
| **ABCB1 (MDR1)** | 3.28 | 2.29 | 3.84 | 10.0 | 4.57 | 8.06 | 3.38 | 1.14 | 4.77 | 3.09 | 6.84 | 2.60 | 6.79 | 4.50 | 3.87 | 2.33 | 6.39 | 4.06 | 2.54 | 1.76 | 3.59 | 2.23 | 4.18 | 2.16 | 52 | 22 |
| **ABCB2 (TAP1)** | 0.40 | 0.39 | 0.48 | 1.20 | 0.67 | 0.11 |  |  | 0.69 |  | 0.17 |  | 0.17 |  | 0.54 |  | 0.67 | 0.77 |  |  |  | 0.11 | 0.49 | 0.30 | 62 | 13 |
| **ABCB6 (MTABC3)** | 0.11 | 0.10 |  |  |  | 0.21 |  |  |  |  |  |  |  |  |  |  |  |  |  |  |  | 0.05 | 0.12 | 0.06 | 49 | 4 |
| **ABCC9 (SUR2)** |  |  |  |  |  |  |  |  |  |  | 0.06 |  |  |  |  |  |  |  |  |  |  |  | 0.06 |  |  | 1 |
| **ABCD3 (PMP70)** |  | 0.44 |  |  |  |  |  |  | 0.60 |  |  |  | 0.43 |  | 0.59 |  | 0.16 | 0.14 |  |  | 0.40 | 0.13 | 0.36 | 0.18 | 50 | 8 |
| **ABCE1 (RLI)** |  | 0.14 | 0.14 | 0.24 | 0.22 |  |  |  | 0.21 |  |  |  |  |  |  |  |  |  |  |  |  | 0.12 | 0.18 | 0.05 | 26 | 6 |
| **ABCF1 (ABC50)** |  | 0.12 |  |  | 0.14 |  |  |  |  |  |  |  |  |  |  |  |  |  |  |  |  |  | 0.13 | 0.01 | 8 | 2 |
| **ABCF2 (HUSSY-18)** |  | 0.19 | 0.32 | 0.26 | 0.22 | 0.20 | 0.20 |  | 0.22 | 0.17 |  | 0.16 | 0.27 | 0.19 | 0.25 |  | 0.16 | 0.14 | 0.12 | 0.12 | 0.11 | 0.17 | 0.19 | 0.06 | 29 | 18 |
| **ABCG2 (BCRP)** | 0.94 | 1.41 | 2.82 | 4.75 | 2.74 | 2.85 | 1.58 | 0.77 | 2.54 | 1.69 | 4.13 | 1.06 | 3.51 | 3.58 | 1.19 | 1.67 | 3.80 | 2.16 | 1.88 | 1.31 | 2.65 | 1.61 | 2.30 | 1.09 | 48 | 22 |

**Supplementary Table 7:** ABC transporter abundance per milligram totalprotein in individual kidney samples. Individual measurements, means, standard deviation values and %CV in individual values are presented. The number n is the number of kidney samples in which a protein was quantified.

| **Transporter** | **Kidney abundance (pmol mg^-1^)** | | | | | | | | | | | | | | | | | | | | | | | |
| --- | --- | --- | --- | --- | --- | --- | --- | --- | --- | --- | --- | --- | --- | --- | --- | --- | --- | --- | --- | --- | --- | --- | --- | --- |
|  | **K11** | **K12** | **K13** | **K14** | **K15** | **K16** | **K17** | **K18** | **K19** | **K20** | **K21** | **K22** | **K23** | **K24** | **K25** | **K26** | **K27** | **K28** | **K29** | **K30** | **Mean** | **SD** | **%CV** | **n** |
| **ABCA8** | 0.55 | 0.53 | 0.06 | 0.12 | 0.08 | 0.07 | 0.08 | 0.07 | 0.05 | 0.09 | 0.05 | 0.18 | 0.52 | 0.08 | 0.57 | 0.39 | 0.23 | 0.53 | 0.05 | 0.06 | 0.22 | 0.2 | 93 | 20 |
| **ABCB1 (MDR1)** | 2.95 | 5.68 | 4.50 | 1.84 | 4.36 | 4.56 | 7.16 | 3.62 | 3.76 | 6.01 | 2.97 | 2.99 | 3.87 | 2.44 | 2.04 | 5.00 | 3.77 | 3.52 | 3.37 | 3.31 | 3.89 | 1.3 | 33 | 20 |
| **ABCB2 (TAP1)** | 0.91 | 0.08 | 0.06 | 0.23 |  | 0.15 | 0.07 | 0.10 |  | 0.59 | 0.34 |  | 0.05 | 0.11 | 0.07 |  | 0.17 | 0.06 | 0.05 | 0.04 | 0.19 | 0.23 | 119 | 16 |
| **ABCB3 (TAP2)** | 0.35 |  |  | 0.25 |  | 0.24 | 0.05 | 0.10 |  |  | 0.21 |  |  | 0.23 | 0.04 |  | 0.16 |  |  | 0.03 | 0.17 | 0.1 | 61 | 10 |
| **ABCB6 (MTABC3)** |  | 0.15 | 0.07 | 0.08 | 0.16 | 0.11 | 0.37 | 0.07 | 0.22 | 0.18 | 0.10 |  | 0.79 | 0.05 | 0.14 |  | 0.19 | 0.14 | 0.14 | 0.12 | 0.18 | 0.17 | 93 | 17 |
| **ABCB8 (M-ABC1)** | 0.02 | 0.08 | 0.04 | 0.01 | 0.09 |  | 0.18 | 0.01 |  |  | 0.01 | 0.04 |  | 0.38 | 0.04 | 0.08 | 0.11 | 0.12 |  | 0.05 | 0.08 | 0.09 | 109 | 15 |
| **ABCC1 (MRP1)** | 0.06 |  |  |  |  |  |  |  |  |  |  |  |  |  |  |  |  |  |  |  | 0.06 | 0 | 0 | 1 |
| **ABCC2 (MRP2)** |  | 0.11 | 0.08 | 0.00 | 0.14 | 0.02 | 0.36 | 0.08 | 0.07 |  | 0.06 | 0.18 | 0.04 | 0.09 | 0.08 | 0.07 | 0.13 | 0.03 | 0.16 | 0.08 | 0.10 | 0.08 | 79 | 18 |
| **ABCC3 (MRP3)** | 0.07 |  |  |  |  | 0.01 |  |  |  |  | 0.01 |  |  |  | 0.13 | 0.19 |  |  |  |  | 0.08 | 0.07 | 86 | 5 |
| **ABCC4 (MRP4)** | 0.09 | 0.15 | 0.12 | 0.08 | 0.12 | 0.28 | 0.37 | 0.25 | 0.06 | 0.13 | 0.10 | 0.10 | 0.13 | 0.04 | 0.09 | 0.08 | 0.26 | 0.20 | 0.11 | 0.20 | 0.15 | 0.08 | 56 | 20 |
| **ABCC6 (MRP6)** |  | 0.29 | 0.02 | 0.02 | 0.13 | 0.04 | 1.66 | 0.04 | 0.20 |  | 0.06 | 0.11 | 0.16 | 0.35 | 0.09 | 0.13 | 0.20 | 0.16 | 0.23 | 0.17 | 0.23 | 0.36 | 159 | 18 |
| **ABCD3 (PMP70)** | 0.45 | 1.83 | 1.44 | 0.22 | 1.67 | 1.03 | 1.99 | 1.24 | 0.78 | 1.13 | 0.59 | 0.85 | 0.41 | 1.61 | 0.37 | 1.00 | 1.68 | 1.06 | 0.79 | 1.20 | 1.07 | 0.51 | 48 | 20 |
| **ABCD4 (PXMP1L)** |  |  | 0.02 |  |  | 0.02 |  |  |  |  |  |  |  |  | 0.04 |  |  |  |  |  | 0.03 | 0.01 | 37 | 3 |
| **ABCE1 (RLI)** | 1.68 |  | 0.38 |  | 0.14 | 0.50 | 0.26 | 0.25 | 0.09 | 0.78 | 0.68 | 0.56 | 0.18 |  | 0.16 |  | 0.08 |  | 0.11 | 0.16 | 0.40 | 0.4 | 101 | 15 |
| **ABCF1 (ABC50)** | 0.14 | 0.21 | 0.19 | 0.12 | 0.10 | 0.10 | 0.04 | 0.22 | 0.04 | 0.10 | 0.32 | 0.21 | 0.10 | 0.24 | 0.19 | 0.03 |  | 0.08 | 0.10 | 0.10 | 0.14 | 0.08 | 55 | 19 |
| **ABCF2 (HUSSY-18)** |  |  |  |  | 0.03 | 0.13 |  | 0.01 |  |  | 0.01 | 0.02 |  |  |  |  |  |  | 0.07 | 0.09 | 0.05 | 0.04 | 82 | 7 |
| **ABCF3** |  | 0.23 | 0.05 | 0.02 | 0.10 |  | 0.26 | 0.12 | 0.17 | 0.11 | 0.06 | 0.09 |  |  | 0.16 |  | 0.08 | 0.30 | 0.08 | 0.28 | 0.14 | 0.09 | 62 | 15 |

**Supplementary Table 8:** ABC transporter abundance per milligram total protein in individual skin samples. Individual measurements, means, standard deviation values and %CV in individual values are presented. The number n is the number of skin samples in which a protein was quantified.

| **Transporter** | **Skin abundance (pmol mg^-1^)** | | | | | | | | | | |
| --- | --- | --- | --- | --- | --- | --- | --- | --- | --- | --- | --- |
|  | **S07** | **S52** | **S60** | **S56** | **S57** | **S58** | **LS** | **Mean** | **SD** | **%CV** | **n** |
| **ABCA6** |  |  | 0.09 |  |  |  |  | 0.09 |  |  | 1 |
| **ABCA8** |  | 0.01 | 0.80 |  |  |  |  | 0.41 | 0.40 | 97 | 2 |
| **ABCB2 (TAP1)** |  |  | 0.15 |  |  |  |  | 0.15 |  |  | 1 |
| **ABCB3 (TAP2)** |  |  | 0.12 |  |  |  |  | 0.12 |  |  | 1 |
| **ABCB6 (MTABC3)** |  |  | 0.01 |  |  |  |  | 0.01 |  |  | 1 |
| **ABCC12 (MRP9)** | 0.12 |  | 0.07 |  |  |  | 0.08 | 0.09 | 0.02 | 23 | 3 |
| **ABCD3 (PMP70)** |  |  | 0.07 |  |  |  |  | 0.07 |  |  | 1 |
| **ABCE1 (RLI)** |  |  |  | 0.36 |  |  | 0.10 | 0.23 | 0.13 | 57 | 2 |

**Supplementary Table 9:** ABC transporter abundance in pmol per milligram total protein in individual paediatric liver set 1 samples.

| **Transporter** | **Paediatric Liver set 1 abundance (pmol mg^-1^)** | | | | | | | | | | | | | | | | | | | | | | | | | | | | | | | | | | | | |  |
| --- | --- | --- | --- | --- | --- | --- | --- | --- | --- | --- | --- | --- | --- | --- | --- | --- | --- | --- | --- | --- | --- | --- | --- | --- | --- | --- | --- | --- | --- | --- | --- | --- | --- | --- | --- | --- | --- | --- |
| **Sample YE** | **01** | **02** | **03** | **04** | **05** | **06** | **07** | **08** | **09** | **10** | **11** | **12** | | **13** | | **14** | | **15** | | **16** | | **17** | | **18** | | **19** | | **20** | | **Mean** | | **SD** | | **%CV** | | **n** | |  |
| **ABCA1 (ABC1)** |  |  | 0.02 |  |  | 0.02 | 0.02 |  | 0.03 |  |  | |  | |  | |  | |  | |  | |  | |  | |  | |  | | 0.02 | | 0.003 | | 13 | | 4 | |
| **ABCA2 (ABC2)** |  |  |  |  |  |  |  | 0.02 |  |  |  | | 0.01 | |  | |  | |  | | 0.02 | |  | |  | | 0.01 | |  | | 0.01 | | 0.003 | | 17 | | 4 | |
| **ABCA6** | 0.97 | 0.34 | 1.27 | 0.33 | 1.00 | 0.93 | 0.51 | 0.95 | 0.60 | 2.84 | 1.06 | | 0.96 | | 1.24 | | 1.19 | | 2.04 | | 1.71 | | 1.80 | | 0.86 | | 0.54 | | 1.01 | | 1.11 | | 0.59 | | 54 | | 20 | |
| **ABCA8** |  | 0.03 | 0.01 |  |  | 0.16 |  | 0.13 | 0.01 | 0.36 |  | | 0.01 | |  | | 0.14 | | 0.06 | | 0.64 | | 0.19 | |  | | 0.06 | | 0.52 | | 0.18 | | 0.20 | | 110 | | 13 | |
| **ABCB1 (MDR1)** | 0.08 | 0.33 | 0.14 | 0.79 | 0.30 | 0.06 | 0.07 | 0.16 | 0.67 | 0.18 | 0.08 | | 0.06 | | 0.05 | | 0.48 | | 0.31 | | 0.06 | | 0.09 | | 0.05 | | 0.03 | | 0.05 | | 0.20 | | 0.21 | | 105 | | 20 | |
| **ABCB2 (TAP1)** | 0.06 | 0.22 | 0.23 | 0.89 | 0.22 | 0.20 | 0.49 | 0.04 | 0.42 | 0.23 | 1.09 | | 0.23 | | 0.30 | | 0.32 | | 0.99 | | 0.83 | | 0.79 | | 0.17 | | 0.36 | | 0.30 | | 0.42 | | 0.31 | | 74 | | 20 | |
| **ABCB3 (TAP2)** | 0.04 | 0.08 | 0.47 | 1.01 | 0.19 | 0.09 | 0.55 |  | 0.31 | 0.03 | 1.31 | | 0.04 | | 0.36 | | 0.22 | | 1.05 | | 0.44 | | 1.39 | | 0.18 | | 0.64 | | 0.49 | | 0.47 | | 0.42 | | 90 | | 19 | |
| **ABCB4 (MDR3)** |  | 0.01 | 0.04 | 0.04 | 0.02 | 0.01 | 0.01 | 0.10 | 0.11 | 0.05 | 0.004 | | 0.02 | | 0.01 | | 0.06 | | 0.01 | | 0.01 | |  | | 0.01 | | 0.02 | | 0.01 | | 0.03 | | 0.03 | | 106 | | 18 | |
| **ABCB6 (MTABC3)** |  | 0.07 | 0.03 |  | 1.41 |  | 0.28 | 0.02 | 3.02 | 0.30 | 0.34 | | 0.44 | | 0.38 | | 0.30 | | 0.03 | | 0.22 | | 0.17 | | 0.45 | | 0.04 | | 0.07 | | 0.44 | | 0.72 | | 162 | | 17 | |
| **ABCB7 (ABC7)** |  |  |  |  | 0.02 |  | 0.04 |  | 1.23 | 0.46 | 0.41 | | 0.29 | | 0.06 | | 0.86 | | 0.81 | | 0.52 | | 0.26 | | 0.24 | | 1.10 | | 0.08 | | 0.46 | | 0.39 | | 85 | | 14 | |
| **ABCB8 (M-ABC1)** |  |  |  |  |  |  | 0.13 | 0.10 | 1.22 | 0.35 | 0.07 | | 0.45 | | 0.19 | | 0.59 | | 0.28 | | 0.16 | | 0.06 | | 0.25 | | 0.28 | | 0.14 | | 0.31 | | 0.29 | | 95 | | 14 | |
| **ABCB10 (M-ABC2)** |  |  |  |  |  |  | 0.04 |  | 0.33 | 0.09 | 0.12 | | 0.37 | |  | | 0.37 | | 0.21 | | 0.18 | | 0.16 | | 0.07 | | 0.07 | | 0.05 | | 0.17 | | 0.12 | | 69 | | 12 | |
| **ABCB11 (BSEP)** |  | 0.26 | 0.33 | 0.33 | 0.45 | 0.37 | 0.52 | 1.10 | 2.57 | 0.60 | 0.22 | | 0.27 | | 0.19 | | 0.92 | | 0.27 | | 0.75 | | 0.05 | | 0.08 | | 0.09 | | 0.32 | | 0.51 | | 0.56 | | 109 | | 19 | |
| **ABCC2 (MRP2)** |  | 1.40 |  | 0.52 | 0.52 |  | 0.03 | 0.14 | 3.03 |  | 0.03 | | 0.13 | | 0.02 | | 1.05 | |  | |  | |  | | 0.02 | | 0.02 | |  | | 0.58 | | 0.86 | | 150 | | 12 | |
| **ABCC3 (MRP3)** |  | 0.12 |  | 0.03 | 0.03 | 0.01 | 0.02 | 0.03 | 0.03 |  |  | |  | |  | | 0.05 | | 0.13 | |  | |  | |  | |  | |  | | 0.05 | | 0.04 | | 78 | | 9 | |
| **ABCC6 (MRP6)** | 0.01 | 0.25 | 0.04 | 0.05 | 0.08 | 0.06 | 0.04 | 0.19 | 0.68 | 0.50 | 0.06 | | 0.02 | | 0.08 | | 0.24 | | 1.21 | | 0.11 | | 0.02 | | 0.04 | | 0.07 | | 0.04 | | 0.19 | | 0.29 | | 152 | | 20 | |
| **ABCD1 (ALD)** |  |  |  |  |  |  |  |  | 0.89 |  |  | | 0.26 | |  | | 0.04 | | 0.24 | | 0.04 | |  | | 0.03 | |  | |  | | 0.25 | | 0.30 | | 119 | | 6 | |
| **ABCD3 (PMP70)** | 1.59 | 0.87 | 1.54 | 1.75 | 3.36 | 1.21 | 2.42 | 3.09 | 33.27 | 20.69 | 4.67 | | 13.82 | | 3.00 | | 8.66 | | 11.31 | | 15.29 | | 4.30 | | 12.32 | | 9.12 | | 2.86 | | 7.76 | | 8.03 | | 103 | | 20 | |
| **ABCD4 (PXMP1L)** |  |  |  |  | 0.05 |  |  |  |  |  |  | | 0.08 | |  | |  | |  | |  | |  | |  | |  | | 0.06 | | 0.06 | | 0.01 | | 24 | | 3 | |
| **ABCE1 (RLI)** | 1.35 | 2.49 | 1.68 | 3.21 | 2.76 | 0.96 | 0.96 | 0.75 | 0.27 |  |  | | 0.06 | |  | |  | |  | |  | |  | |  | | 0.10 | |  | | 1.33 | | 1.04 | | 78 | | 11 | |
| **ABCF1 (ABC50)** | 0.18 | 0.50 | 0.29 | 0.26 | 0.27 | 0.05 | 0.23 | 0.09 |  |  |  | | 0.03 | |  | |  | | 0.16 | |  | |  | |  | | 0.05 | |  | | 0.19 | | 0.13 | | 68 | | 11 | |
| **ABCF2 (HUSSY-18)** |  | 0.07 |  | 0.09 |  |  | 0.06 |  |  |  |  | |  | |  | |  | | 0.05 | |  | |  | |  | |  | |  | | 0.07 | | 0.02 | | 25 | | 4 | |
| **ABCG2 (BCRP)** |  |  |  | 0.02 |  |  |  | 0.01 |  |  |  | |  | |  | |  | |  | |  | |  | |  | | 0.01 | |  | | 0.01 | | 0.01 | | 58 | | 3 | |
| **ABCG8** |  |  |  |  |  |  |  | 0.05 | 0.09 |  |  | | 0.03 | |  | |  | |  | |  | |  | |  | |  | |  | | 0.06 | | 0.02 | | 42 | | 3 | |

| **Transporter** | **Paediatric Liver set 2 abundance (pmol mg^-1^)** | | | | | | | | | | | | | | | | | | | | | | | | | | |  |
| --- | --- | --- | --- | --- | --- | --- | --- | --- | --- | --- | --- | --- | --- | --- | --- | --- | --- | --- | --- | --- | --- | --- | --- | --- | --- | --- | --- | --- |
|  | **b1**  **i** | **b1**  **ii** | **b1**  **iii** | **b1**  **iv** | **b2**  **v** | **b2**  **vi** | **b2**  **vii** | **b2**  **viii** | **b2**  **ix** | **b2**  **x** | **b3**  **xi** | **b3**  **xii** | **b3**  **xiii** | **b3**  **xiv** | **b3**  **xv** | **b3**  **xvi** | **b3**  **xvii** | **b3**  **xvii** | **b4**  **xx** | **b4**  **xxi** | **b4**  **xxii** | **b4**  **xxiii** | **b4**  **xxiv** | **Mean** | **SD** | **%CV** | **n** | |
| **ABCA3** |  |  |  |  |  | 1.32 |  |  |  |  |  |  |  |  |  |  |  |  |  | 0.17 |  |  |  | 0.74 | 0.57 | 77 | 2 | |
| **ABCA5** |  |  |  |  |  | 0.04 |  |  |  |  |  |  |  |  |  |  |  |  |  |  |  |  |  | 0.04 |  |  | 1 | |
| **ABCA6** |  | 0.03 | 0.53 | 0.19 | 0.63 |  | 0.04 | 0.40 | 0.31 |  |  |  |  |  | 0.14 |  |  | 0.07 |  |  |  |  |  | 0.26 | 0.21 | 80 | 9 | |
| **ABCA8** | 0.03 |  |  | 0.04 |  |  | 0.15 | 0.06 | 0.27 |  |  |  |  |  |  |  |  |  |  |  |  |  |  | 0.11 | 0.09 | 82 |  | |
| **ABCB1**  **(MDR1)** | 0.01 |  | 0.03 | 0.03 | 0.05 |  | 0.02 | 0.03 | 0.02 | 0.01 | 0.02 |  |  | 0.01 |  |  |  | 0.03 |  |  | 0.03 |  |  | 0.02 | 0.01 | 45 | 12 | |
| **ABCB4**  **(MDR3)** | 0.14 | 0.04 | 0.07 | 0.09 | 0.05 | 0.05 | 0.13 | 0.06 | 0.19 | 0.11 | 0.11 | 0.03 | 0.05 | 0.09 |  | 0.05 | 0.03 | 0.05 |  | 0.03 | 0.06 |  | 0.05 | 0.07 | 0.04 | 57 | 20 | |
| **ABCB6** |  |  | 0.31 | 0.05 | 0.12 | 0.07 |  | 0.33 | 0.09 | 0.05 | 0.43 |  |  |  |  |  |  | 0.25 |  |  | 0.41 |  |  | 0.21 | 0.15 | 69 | 10 | |
| **ABCB11**  **(BSEP)** | 0.05 | 0.02 | 0.06 | 0.02 | 0.01 |  | 0.25 | 0.04 | 0.30 | 0.08 | 0.07 | 0.14 |  | 0.07 | 0.14 | 0.04 |  | 0.21 |  |  | 0.05 | 0.06 |  | 0.09 | 0.08 | 88 | 17 | |
| **ABCC2**  **(MRP2)** |  |  | 0.03 |  |  |  | 0.01 |  |  |  |  |  |  |  |  |  |  |  |  |  |  |  |  | 0.02 | 0.01 | 42 | 2 | |
| **ABCC3**  **(MRP3)** | 0.05 |  |  |  |  | 0.002 | 0.49 |  | 0.18 |  |  |  |  |  |  |  |  |  |  |  |  |  |  | 0.18 | 0.19 | 105 | 4 | |
| **ABCC6**  **(MRP6)** | 0.02 | 0.02 | 0.29 | 0.07 | 0.16 |  | 0.03 | 0.09 | 0.02 | 0.02 |  |  |  |  | 0.09 |  |  | 0.02 |  |  | 0.04 |  |  | 0.07 | 0.08 | 108 | 12 | |
| **ABCD3** | 0.58 | 0.94 | 1.52 | 0.89 | 0.12 |  | 4.83 | 0.24 | 4.39 |  | 0.44 | 1.06 | 0.32 | 0.16 | 3.14 | 0.42 | 1.50 | 0.79 |  |  |  | 3.38 |  | 1.45 | 1.47 | 101 | 17 | |
| **ABCE1** | 0.04 | 0.60 | 1.57 | 0.45 | 1.97 |  | 0.13 | 2.41 | 0.38 |  |  |  | 0.26 |  |  | 0.27 |  | 0.87 | 0.29 |  | 0.61 |  |  | 0.76 | 0.72 | 95 |  | |
| **ABCF1** |  | 0.17 | 0.59 |  | 0.89 |  | 0.02 | 0.55 |  |  |  |  |  |  |  |  |  |  |  |  |  |  |  | 0.44 | 0.31 | 70 | 5 | |
| **ABCF2** |  |  | 0.04 |  | 0.50 |  |  | 0.34 |  |  |  |  |  |  |  |  |  |  |  |  |  |  |  | 0.29 | 0.19 | 66 | 3 | |
| **ABCG2**  **(BCRP)** |  | 0.01 | 0.01 |  | 0.01 |  |  |  |  |  |  |  |  |  |  |  | 0.06 |  |  |  |  |  |  | 0.02 | 0.02 | 85 | 4 | |

**Supplementary Table 10:** ABC transporter abundance in pmol per milligram total protein in individual paediatric liver set 2 samples.

| **Trans-porter/ Sample** | **1** | **2** | **3** | **4** | **5** | **9** | **10** | **11** | **12** | **14** | **15** | **16** | **17** | **18** | **19** | **20** | **21** | **22** | **23** | **24** | **25** | **26** | **27** | **28** | **29** | **Mean** | **SD** | **%CV** | **n** |
| --- | --- | --- | --- | --- | --- | --- | --- | --- | --- | --- | --- | --- | --- | --- | --- | --- | --- | --- | --- | --- | --- | --- | --- | --- | --- | --- | --- | --- | --- |
| **ABCA6** | 0.35 | 0.26 | 0.36 | 0.19 | 0.35 | 0.24 |  | 0.37 | 0.59 | 0.41 | 0.54 | 0.98 | 0.37 | 0.74 | 0.76 | 0.52 | 0.35 | 0.55 | 1.04 | 0.62 | 0.48 | 0.38 | 0.61 | 0.75 | 0.36 | 0.51 | 0.22 | 43 | 24 |
| **ABCA8** | 0.03 | 0.01 | 0.02 |  |  | 0.002 |  |  | 0.01 | 0.005 | 0.004 | 0.03 | 0.01 | 0.07 | 0.08 | 0.01 | 0.003 | 0.39 | 0.17 | 0.01 | 0.004 | 0.01 | 0.01 | 0.04 | 0.004 | 0.04 | 0.09 | 195 | 21 |
| **ABCB1** | 0.42 | 1.37 | 0.58 | 0.40 | 0.44 | 0.18 | 1.93 | 0.32 | 0.32 | 1.17 | 0.29 | 0.29 | 0.44 | 0.16 |  | 0.11 | 0.15 | 0.20 | 0.17 | 0.27 | 0.24 | 0.31 | 0.84 |  | 0.14 | 0.47 | 0.44 | 95 | 23 |
| **ABCB2** |  |  |  | 0.03 | 0.09 | 0.02 |  |  |  |  |  | 0.03 |  | 0.16 |  |  |  |  |  |  | 0.01 |  | 0.03 |  | 0.17 | 0.07 | 0.06 | 90 | 8 |
| **ABCB3** | 0.02 |  | 0.02 | 0.24 | 0.05 | 0.02 |  |  | 0.02 |  | 0.03 |  |  | 1.43 | 0.03 |  |  |  |  | 0.05 | 0.02 | 0.01 | 0.03 |  | 0.11 | 0.15 | 0.36 | 243 | 14 |
| **ABCB4** | 0.49 | 0.99 | 1.58 | 1.31 |  |  |  | 4.19 | 0.72 |  |  | 0.22 |  | 2.32 |  | 1.22 | 0.89 | 3.82 | 5.08 |  | 0.12 | 0.69 | 0.83 | 1.08 | 0.17 | 1.51 | 1.44 | 95 | 17 |
| **ABCB7** |  |  |  |  | 0.06 | 0.07 |  |  |  |  | 0.07 |  |  | 0.07 |  | 0.06 | 0.08 | 0.08 |  | 0.07 | 0.01 |  | 0.04 |  | 0.05 | 0.06 | 0.02 | 32 | 11 |
| **ABCB8** |  |  |  |  |  |  |  |  |  |  | 0.04 |  |  | 0.05 | 0.03 |  |  |  | 0.03 |  |  |  | 0.05 |  | 0.10 | 0.05 | 0.02 | 50 | 6 |
| **ABCB11** | 0.46 | 0.59 | 0.63 | 0.61 | 0.23 | 0.39 | 1.12 | 0.42 | 0.39 | 0.72 | 0.15 | 0.17 | 0.67 | 0.09 | 0.04 | 0.18 | 0.41 | 0.15 | 0.14 | 0.26 | 0.33 | 0.40 | 0.48 | 0.09 | 0.19 | 0.37 | 0.25 | 66 | 25 |
| **ABCC2** |  | 0.01 |  |  |  | 0.01 |  |  |  |  |  | 0.02 |  | 0.03 |  |  |  | 0.02 | 0.45 |  |  |  | 0.01 |  | 0.01 | 0.07 | 0.14 | 211 | 8 |
| **ABCC3** | 0.21 | 0.33 | 0.36 | 0.21 | 0.07 | 0.21 | 0.27 | 0.23 | 0.24 | 0.31 | 0.06 | 0.08 | 0.16 | 0.10 |  | 0.05 | 0.13 | 0.10 | 0.05 | 0.08 | 0.15 | 0.13 | 0.18 | 0.03 | 0.06 | 0.15 | 0.09 | 61 | 24 |
| **ABCC4** | 0.20 | 0.33 | 0.45 | 0.20 | 0.08 | 0.13 | 0.31 | 0.35 | 0.25 | 0.41 | 0.08 |  | 0.19 | 0.03 |  | 0.05 | 0.26 | 0.03 |  | 0.05 | 0.19 | 0.15 | 0.26 |  |  | 0.20 | 0.12 | 63 | 20 |
| **ABCC6** | 1.09 | 1.12 | 1.08 | 0.59 | 0.21 | 0.65 | 1.24 | 1.14 | 0.88 | 0.83 | 0.52 | 0.56 | 0.76 | 0.45 | 0.26 | 0.55 | 1.26 | 0.71 | 0.52 |  | 0.68 | 0.70 | 0.80 | 0.37 | 0.52 | 0.73 | 0.29 | 40 |  |
| **ABCD1** |  |  |  |  |  |  |  |  |  |  |  |  |  | 0.31 |  |  |  | 0.06 | 0.02 |  |  |  |  |  |  | 0.13 | 0.13 | 101 | 3 |
| **ABCD3** | 0.44 | 0.51 | 0.69 | 0.41 | 0.08 | 1.85 |  | 0.64 | 0.24 | 2.07 | 0.55 | 1.92 | 0.42 | 2.60 | 0.36 | 1.38 | 1.90 | 1.35 | 1.32 | 0.68 | 0.91 | 0.31 | 1.90 | 0.81 | 1.90 | 1.05 | 0.71 | 68 | 24 |
| **ABCD4** |  |  | 0.02 |  |  |  |  |  |  |  |  | 0.04 |  | 0.05 | 0.04 |  | 0.03 |  |  |  |  |  |  |  | 0.03 | 0.04 | 0.01 | 28 | 6 |
| **ABCE1** | 0.03 | 0.05 | 0.17 | 0.09 | 0.20 | 0.30 |  |  | 0.03 | 0.05 | 1.27 | 0.30 | 0.19 | 0.80 | 0.69 | 0.41 |  | 0.26 | 0.37 | 0.48 | 0.14 | 0.13 | 0.34 | 0.41 | 0.96 | 0.35 | 0.32 | 91 |  |
| **ABCF1** |  |  |  |  | 0.03 |  |  |  | 0.27 |  |  | 0.49 |  |  | 0.06 |  |  |  |  |  | 0.14 | 0.13 |  | 0.03 | 0.50 | 0.21 | 0.18 | 88 | 8 |
| **ABCF2** |  |  |  |  |  |  |  |  |  |  | 0.01 |  |  |  | 0.10 |  |  |  |  |  |  |  | 0.03 |  |  | 0.05 | 0.04 | 79 |  |
| **ABCG2** | 0.90 | 1.56 | 1.50 | 1.02 | 0.31 | 0.74 | 2.06 | 0.99 | 1.13 | 1.68 | 0.54 | 0.66 | 0.97 | 0.44 |  | 0.37 | 0.79 | 0.47 | 0.66 | 0.60 | 0.75 | 0.69 | 0.73 | 0.22 | 0.38 | 0.84 | 0.46 | 54 | 24 |
| **ABCG8** |  |  |  |  |  |  |  |  |  |  |  | 0.01 |  | 0.02 |  |  |  | 0.02 | 0.02 |  |  |  |  |  |  | 0.02 | 0.004 | 19 | 4 |

**Supplementary Table 11:** ABC transporter abundance in pmol per mgtotal protein in biliary atresia liver samples

**Supplementary Table 12:** ABC transporter abundance in pmol per mg total protein in adult liver samples set 1

| **Trans-porter/ Sample** | **01** | **02** | **06** | **08** | **09** | **100** | **108** | **117** | **11** | **25** | **35** | **38** | **41** | **48** | **71** | **72** | **73** | **74** | **75** | **76** | **77** | **78** | **79** | **80** | **89** | **90** | **91** | **Mean** | **SD** | **%CV** | **n** |
| --- | --- | --- | --- | --- | --- | --- | --- | --- | --- | --- | --- | --- | --- | --- | --- | --- | --- | --- | --- | --- | --- | --- | --- | --- | --- | --- | --- | --- | --- | --- | --- |
| **ABCA1** |  | 0.17 | 0.02 | 0.06 | 0.07 | 0.04 | 0.01 | 0.03 | 0.05 | 0.02 | 0.01 | 0.04 | 0.01 |  | 0.03 | 0.04 | 0.04 | 0.02 | 0.01 | 0.05 | 0.03 | 0.05 | 0.05 | 0.03 | 0.18 | 0.04 | 0.01 | 0.04 | 0.04 | 99 | 25 |
| **ABCA2** |  | 0.04 | 0.02 | 0.01 | 0.01 | 0.04 |  | 0.02 | 0.01 | 0.02 |  | 0.03 | 0.05 |  | 0.04 | 0.04 | 0.02 |  | 0.03 | 0.03 | 0.00 | 0.04 | 0.06 |  | 0.05 | 0.02 | 0.01 | 0.03 | 0.02 | 57 | 21 |
| **ABCA6** | 0.35 | 0.30 | 1.31 | 0.82 | 0.91 | 0.52 | 0.59 | 0.55 | 0.52 | 0.85 | 0.73 | 0.53 | 1.28 | 0.13 | 1.19 | 2.18 | 1.08 | 0.58 | 0.24 | 0.68 | 0.30 | 1.14 | 0.63 | 0.06 | 1.27 | 0.82 | 0.28 | 0.74 | 0.45 | 62 | 27 |
| **ABCA8** |  |  | 0.01 | 0.04 | 0.02 | 0.01 |  | 0.04 | 0.08 |  |  |  | 0.06 | 0.01 | 0.07 | 0.18 | 0.06 |  | 0.01 | 0.01 |  | 0.01 | 0.23 | 0.01 | 0.04 | 0.11 |  | 0.06 | 0.06 | 111 | 18 |
| **ABCB1** | 0.09 | 0.19 | 0.07 | 0.44 | 0.81 | 0.25 | 0.21 | 0.20 | 0.25 | 0.38 | 0.03 | 0.55 | 0.22 | 0.29 | 0.47 | 0.19 | 0.06 | 0.07 | 0.12 | 0.14 | 0.12 | 0.12 | 0.27 | 0.04 | 0.16 | 0.11 | 0.02 | 0.22 | 0.18 | 82 | 27 |
| **ABCB2** | 0.02 | 1.02 |  | 0.24 |  | 0.10 | 0.03 | 0.23 | 0.08 | 0.04 | 0.03 | 0.21 | 0.04 | 0.05 | 0.04 | 0.02 | 0.13 | 0.03 | 0.03 |  | 0.03 | 0.03 | 0.05 | 0.03 | 0.11 | 0.24 | 0.04 | 0.12 | 0.20 | 168 | 24 |
| **ABCB3** | 0.31 | 0.95 | 0.22 | 0.19 | 0.53 | 0.73 | 0.07 | 0.46 | 0.05 | 0.14 | 0.23 | 0.50 | 0.10 | 0.15 | 0.35 | 0.07 | 0.08 | 0.10 | 0.36 | 0.15 | 0.15 | 0.23 | 0.16 | 0.29 | 0.12 | 0.22 | 0.20 | 0.26 | 0.21 | 79 | 27 |
| **ABCB4** | 0.004 | 0.04 | 0.01 | 0.03 | 0.11 | 0.02 | 0.01 | 0.03 | 0.08 | 0.01 | 0.00 | 0.16 | 0.04 | 0.01 | 0.00 | 0.01 | 0.01 | 0.01 | 0.01 | 0.01 | 0.05 | 0.01 | 0.02 | 0.00 | 0.02 | 0.01 | 0.00 | 0.03 | 0.04 | 139 | 27 |
| **ABCB5** |  | 0.001 | 0.001 |  | 0.03 |  |  |  |  |  |  |  |  |  |  |  |  |  | 0.001 |  |  |  |  |  |  |  |  | 0.01 | 0.01 | 155 | 4 |
| **ABCB6** | 0.09 | 0.85 | 0.09 | 0.08 | 0.02 | 0.03 |  | 0.15 | 0.02 | 0.23 | 0.08 | 0.13 |  | 0.01 | 0.02 | 0.04 | 0.35 | 0.19 | 0.58 | 0.04 |  | 0.05 |  | 0.10 | 0.26 | 0.10 | 0.12 | 0.16 | 0.20 | 124 | 23 |
| **ABCB7** | 0.08 | 0.27 | 0.69 | 0.36 | 0.49 | 0.31 | 0.16 | 0.40 | 0.42 | 0.26 | 0.93 | 1.37 | 0.42 | 0.68 | 0.13 | 0.32 | 0.62 | 0.11 | 0.12 | 0.44 | 0.26 | 0.51 | 0.22 | 0.83 | 0.42 | 0.70 | 0.28 | 0.44 | 0.29 | 65 | 27 |
| **ABCB8** | 0.02 | 0.20 | 0.14 | 0.08 | 0.04 | 0.06 | 0.06 | 0.30 | 0.04 | 0.04 | 0.31 | 0.61 | 0.05 | 0.12 | 0.04 | 0.14 | 0.06 | 0.04 | 0.07 | 0.09 | 0.01 | 0.13 | 0.02 | 0.34 | 0.03 | 0.04 | 0.03 | 0.11 | 0.13 | 115 | 27 |
| **ABCB10** | 0.05 | 0.33 | 0.30 | 0.17 | 0.15 | 0.16 | 0.04 | 0.48 | 0.06 | 0.17 | 0.35 | 0.87 | 0.06 | 0.59 | 0.07 | 0.11 | 0.12 | 0.07 | 0.10 | 0.28 | 0.05 | 0.58 | 0.11 | 0.26 | 0.17 | 0.16 | 0.14 | 0.22 | 0.20 | 89 | 27 |
| **ABCB11** | 0.05 | 0.28 | 0.22 | 0.28 | 0.18 | 0.25 | 0.27 | 0.16 | 0.35 | 0.05 | 0.04 | 0.24 | 0.30 | 0.02 | 0.26 | 0.15 | 0.07 | 0.05 | 0.22 | 0.32 | 0.19 | 0.13 | 0.21 | 0.13 | 0.20 | 0.10 | 0.02 | 0.18 | 0.10 | 56 | 27 |
| **ABCC2** |  | 0.02 | 0.003 | 0.04 | 0.02 |  | 0.06 | 0.06 | 0.06 | 0.01 | 0.02 | 0.30 | 0.03 | 0.00 | 0.04 | 0.04 | 0.00 | 0.01 | 0.09 | 0.01 | 0.03 | 0.00 | 0.01 | 0.09 | 0.02 | 0.05 |  | 0.04 | 0.06 | 143 | 24 |
| **ABCC3** | 0.01 | 0.26 | 0.01 |  | 0.12 | 0.02 | 0.02 | 0.09 | 0.07 | 0.04 | 0.22 | 0.18 | 0.30 | 0.04 | 0.08 | 0.01 | 0.06 | 0.06 | 0.04 |  | 0.06 | 0.01 | 0.10 | 0.01 | 0.10 | 0.05 | 0.04 | 0.08 | 0.08 | 98 | 25 |
| **ABCC6** | 0.28 | 0.15 | 0.17 | 0.24 | 0.20 | 0.31 | 0.16 | 0.35 | 0.56 | 0.14 | 0.31 | 0.34 | 0.19 | 0.05 | 0.21 | 0.66 | 0.24 | 0.17 | 0.08 | 0.10 | 0.15 | 0.48 | 0.38 | 0.29 | 0.29 | 0.28 | 0.29 | 0.26 | 0.14 | 52 | 27 |
| **ABCD1** | 0.02 | 0.67 | 0.11 | 0.26 | 0.20 | 0.25 | 0.15 | 0.22 | 0.22 | 0.13 | 0.51 | 0.42 | 0.17 | 0.15 | 0.06 |  | 0.04 | 0.08 | 0.12 | 0.20 | 0.09 | 0.13 | 0.13 | 0.12 | 0.08 | 0.15 | 0.25 | 0.19 | 0.14 | 75 | 26 |
| **ABCD3** | 7.2 | 18.3 | 7.8 | 14.4 | 12.8 | 21.2 | 10.0 | 19.3 | 15.5 | 11.5 | 19.8 | 18.6 | 14.9 | 22.5 | 6.9 | 10.5 | 12.1 | 9.9 | 12.8 | 13.6 | 13.6 | 9.7 | 11.9 | 8.7 | 7.4 | 11.4 | 14.9 | 13 | 4.35 | 33 | 27 |
| **ABCD4** | 0.07 | 0.08 | 0.02 | 0.08 |  |  | 0.10 | 0.19 | 0.07 | 0.17 | 0.02 | 0.002 | 0.15 | 0.04 | 0.02 | 0.04 | 0.05 | 0.01 | 0.03 |  | 0.03 | 0.03 | 0.03 | 0.04 | 0.24 | 0.00 | 0.03 | 0.07 | 0.06 | 94 | 24 |
| **ABCE1** |  |  |  | 0.01 |  |  |  |  | 0.01 |  | 0.01 |  |  |  | 0.01 |  |  |  |  |  |  | 0.01 |  | 0.01 |  | 0.01 |  | 0.01 | 0.002 | 17 | 7 |
| **ABCG8** |  | 0.03 | 0.01 |  |  |  |  | 0.03 |  |  | 0.01 | 0.04 |  |  |  |  | 0.01 |  |  |  |  | 0.01 | 0.02 | 0.01 |  |  | 0.01 | 0.02 | 0.01 | 71 | 10 |

**Supplementary Table 13:** ABC transporter abundance pmol per milligram total protein in individual adult livers samples set 2.

| **Trans-porter/ Sample** | **BA01** | **02** | **03** | **04** | **05** | **06** | **07** | **08** | **09** | **10** | **11** | **12** | **13** | **14** | **15** | **16** | **17** | **18** | **19** | **20** | **21** | **22** | **23** | **24** | **25** | **26** | **27** | **28** | **29** |
| --- | --- | --- | --- | --- | --- | --- | --- | --- | --- | --- | --- | --- | --- | --- | --- | --- | --- | --- | --- | --- | --- | --- | --- | --- | --- | --- | --- | --- | --- |
| **ABCA6** | 0.09 | 0.45 | 0.65 | 0.68 | 0.63 | 0.89 | 0.17 | 1.12 | 0.82 | 0.43 | 2.28 | 1.03 | 0.16 | 1.01 | 1.34 | 0.50 | 0.72 | 1.21 | 0.95 | 0.52 | 0.19 | 0.27 | 0.29 | 0.91 | 0.66 | 0.40 | 0.61 | 0.97 |  |
| **ABCA8** |  |  |  |  |  |  |  |  |  |  | 0.03 |  |  | 0.02 | 0.03 |  | 0.03 | 0.05 | 0.10 | 0.09 |  |  |  | 0.04 |  |  |  |  |  |
| **ABCB1** | 0.90 | 0.82 | 0.99 | 0.72 | 0.67 | 0.40 | 0.29 | 0.50 | 0.44 | 0.25 | 0.94 | 1.12 | 1.66 | 0.57 | 0.94 | 0.58 | 0.62 | 1.09 | 0.77 | 0.79 | 1.12 | 0.59 | 0.97 | 1.73 | 0.77 | 0.52 | 1.16 | 0.49 | 1.90 |
| **ABCB2** | 0.05 |  | 0.05 |  |  | 0.11 |  |  | 0.04 | 0.04 |  | 0.13 | 0.29 | 0.12 |  |  | 0.13 |  | 0.34 | 0.26 | 0.20 | 0.45 |  | 0.22 |  |  | 0.06 | 0.07 |  |
| **ABCB3** |  |  |  |  |  |  |  |  |  |  |  |  | 0.003 | 0.003 |  |  | 0.003 |  |  |  |  |  |  |  |  |  |  |  |  |
| **ABCB4** | 0.01 |  |  | 0.02 | 0.03 | 0.14 |  | 0.06 |  |  | 0.01 | 0.08 | 0.39 |  | 0.01 |  | 0.01 |  | 0.10 |  | 0.01 |  | 0.02 | 0.08 |  |  |  |  |  |
| **ABCB6** |  |  |  |  |  |  |  |  |  |  |  |  |  | 0.05 |  |  |  |  |  |  |  |  |  |  |  |  |  |  |  |
| **ABCB7** | 0.11 |  |  |  |  |  |  |  |  |  | 0.22 | 0.42 | 0.16 | 0.06 |  |  |  |  | 0.27 | 0.12 |  | 0.42 | 0.40 | 0.11 |  | 0.06 | 0.15 |  |  |
| **ABCB8** |  |  |  |  | 0.03 |  |  |  |  |  |  |  | 0.03 | 0.09 |  |  | 0.06 |  |  | 0.09 |  |  |  |  |  |  |  |  |  |
| **ABCB10** |  |  |  |  |  |  |  |  |  |  |  | 0.14 | 0.07 | 0.06 |  |  | 0.06 |  | 1.04 |  |  | 0.61 |  |  |  |  | 1.37 | 0.16 |  |
| **ABCB11** | 0.46 | 0.69 | 0.39 | 0.58 | 0.26 | 0.19 | 0.29 | 0.16 | 0.37 | 0.06 | 0.20 | 0.82 | 0.62 | 0.21 | 0.39 | 0.22 | 0.41 | 0.55 | 0.79 | 0.20 | 0.16 | 0.07 | 0.38 | 1.23 | 0.15 |  | 0.25 | 0.19 | 2.18 |
| **ABCC2** |  | 0.18 |  |  |  |  |  |  |  |  |  | 0.07 |  | 0.12 |  |  |  |  | 0.11 |  | 0.04 |  | 0.06 | 0.11 | 0.03 |  |  |  |  |
| **ABCC3** |  |  |  | 0.06 |  |  | 0.04 |  |  |  | 0.05 | 0.06 | 0.12 | 0.08 |  |  |  |  | 0.08 |  | 0.04 |  |  |  |  |  | 0.05 | 0.04 |  |
| **ABCC6** | 0.22 | 0.29 | 0.33 | 0.25 | 0.24 | 0.14 | 0.07 | 0.15 | 0.16 | 0.07 | 0.69 | 0.54 | 0.87 | 0.61 | 0.33 | 0.17 | 0.23 | 0.34 | 0.59 | 0.18 | 0.31 | 0.07 | 0.63 | 0.28 | 0.14 | 0.08 | 0.43 | 0.30 | 0.87 |
| **ABCD1** |  |  |  |  |  |  |  |  |  |  |  |  |  |  |  |  |  |  |  |  |  |  |  |  |  |  |  |  |  |
| **ABCD3** | 3.46 | 2.48 | 4.84 | 2.33 | 1.86 | 1.11 | 2.22 | 0.69 | 0.39 | 0.18 | 2.25 | 6.88 | 9.08 | 1.32 | 3.14 | 2.30 | 2.43 | 1.78 | 3.11 | 2.23 | 5.30 | 0.89 | 2.25 | 3.85 | 4.89 | 0.89 | 7.17 | 2.79 |  |
| **ABCE1** |  |  | 0.10 |  |  |  |  |  | 0.35 |  | 0.43 | 0.10 |  |  | 0.15 |  |  |  |  |  | 0.25 |  | 0.11 | 0.35 |  |  | 0.13 |  |  |
| **ABCF1** |  |  |  |  |  |  | 0.05 |  |  | 0.03 |  |  |  |  |  |  |  |  |  |  |  |  |  |  |  |  |  |  |  |

| **Trans-porter/ Sample** | **ZA01** | **02** | **03** | **04** | **05** | **06** | **07** | **08** | **09** | **10** | **Mean** | **SD** | **%CV** | **n** |
| --- | --- | --- | --- | --- | --- | --- | --- | --- | --- | --- | --- | --- | --- | --- |
| **ABCA6** | 0.21 | 0.44 | 0.97 | 0.96 | 0.26 | 1.26 | 0.82 | 0.56 | 0.94 | 1.07 | 0.70 | 0.43 | 62 | 38 |
| **ABCA8** |  | 0.06 | 0.08 |  |  | 0.14 |  | 0.06 |  | 0.18 | 0.02 | 0.04 | 181 | 13 |
| **ABCB1** | 0.02 |  |  |  |  | 0.08 |  | 0.06 |  | 0.04 | 0.63 | 0.50 | 79 | 33 |
| **ABCB2** |  | 0.86 | 0.54 |  | 0.14 |  |  | 0.52 | 0.10 | 0.11 | 0.12 | 0.19 | 152 | 22 |
| **ABCB3** |  | 0.05 |  |  |  |  |  |  |  | 0.002 | 0.00 | 0.01 | 515 | 5 |
| **ABCB4** | 0.08 | 0.07 |  |  |  | 0.004 |  | 0.003 |  | 0.06 | 0.03 | 0.07 | 225 | 19 |
| **ABCB6** |  |  | 0.22 | 0.10 | 0.18 | 0.19 |  |  | 0.14 |  | 0.02 | 0.06 | 255 | 6 |
| **ABCB7** | 0.11 | 0.37 | 0.25 | 0.23 | 0.07 | 0.09 | 0.13 | 0.17 | 0.09 | 0.31 | 0.11 | 0.13 | 119 | 22 |
| **ABCB8** |  |  | 0.09 |  |  | 0.13 | 0.10 | 0.09 | 0.11 | 0.12 | 0.02 | 0.04 | 175 | 11 |
| **ABCB10** | 0.05 | 0.08 | 0.04 | 0.04 |  |  | 0.04 | 0.12 |  | 0.08 | 0.10 | 0.28 | 274 | 15 |
| **ABCB11** | 0.14 | 0.17 | 0.45 | 0.60 | 0.40 | 0.35 | 0.40 | 0.47 | 0.38 | 0.10 | 0.41 | 0.38 | 92 | 38 |
| **ABCC2** | 0.15 | 0.08 | 0.07 | 0.39 | 0.04 | 0.14 | 0.12 | 0.08 | 0.16 | 0.06 | 0.05 | 0.08 | 150 | 18 |
| **ABCC3** |  |  | 0.10 | 0.08 |  |  |  | 0.05 | 0.04 |  | 0.02 | 0.03 | 147 | 14 |
| **ABCC6** | 0.17 | 0.21 | 0.28 | 0.34 | 0.41 | 0.98 | 0.42 | 0.76 | 0.62 | 0.60 | 0.37 | 0.24 | 65 | 39 |
| **ABCD1** |  | 0.11 |  |  |  |  |  |  |  |  | 0.00 | 0.02 | 616 | 1 |
| **ABCD3** | 7.32 | 16.04 | 12.62 | 6.11 | 3.96 | 8.27 | 6.41 | 5.61 | 5.02 | 5.80 | 4.08 | 3.35 | 82 | 38 |
| **ABCE1** | 0.37 | 0.46 |  | 0.38 | 0.19 | 0.59 |  | 0.27 | 0.02 | 0.12 | 0.11 | 0.16 | 145 | 17 |
| **ABCF1** |  |  |  |  |  | 0.02 |  |  |  |  | 0.00 | 0.01 | 365 | 3 |

**Supplementary Table 13 cont.**

**Supplementary Table 14**: Ontogeny of ABC transporters in the human liver. Abundances are given as mean of non-zero values ± standard error in pmol per mg total protein.

|  | **Feotal** | | **Neonate (< 1m)** | | **Infant (1m-2yr)** | | **Children (2-11yr)** | |  | **Adolescents (12-18yr)** | | **Adults (20-90 yr)** | | **Biliary atresia (2w-5m)** | | |
| --- | --- | --- | --- | --- | --- | --- | --- | --- | --- | --- | --- | --- | --- | --- | --- | --- |
| **Transporter/ Sample** | **N/5** | **mean** | **N/15** | **mean** | **N/7** | **mean** | **N/11** | **mean** |  | **N/4** | **mean** | **N/66** | **mean** | **N/25** | **mean** |  |
| **ABCA1** |  |  | 1 | 0.03 |  |  | 2 | 0.02±0.01 |  | 1 | 0.02±0.01 | 25 | 0.04±0.01 |  |  |  |
| **ABCA2** |  |  |  |  |  |  | 2 | 0.01±0.01 |  | 2 | 0.02±0.01 | 21 | 0.03±0.01 |  |  |  |
| **ABCA6** | 4 | 0.34±0.14 | 7 | 0.63±0.07 | 3 | 1.28±0.23 | 11 | 0.95±0.12 |  | 4 | 1.12±0.28 | 65 | 0.73±0.05 | 24 | 0.51±0.04 |  |
| **ABCA8** | 1 | 0.04 | 6 | 0.15±0.06 | 2 | 0.16±0.02 | 5 | 0.24±0.12 |  | 4 | 0.10±0.02 | 31 | 0.06±0.01 | 21 | 0.04±0.02 |  |
| **ABCB1** | 3 | 0.03±0.01 | 9 | 0.11±0.07 | 5 | 0.13±0.08 | 11 | 0.18±0.06 |  | 4 | 0.14±0.05 | 60 | 0.51±0.06 | 23 | 0.47±0.09 |  |
| **ABCB2** |  |  | 2 | 0.33±0.09 | 3 | 0.42±0.15 | 11 | 0.44±0.10 |  | 4 | 0.40±0.18 | 46 | 0.17±0.03 | 8 | 0.07±0.02 |  |
| **ABCB3** |  |  | 2 | 0.17±0.14 | 3 | 0.60±0.32 | 11 | 0.45±0.12 |  | 3 | 0.59±0.23 | 32 | 0.22±0.04 | 14 | 0.15±0.10 |  |
| **ABCB4** | 5 | 0.06±0.03 | 13 | 0.08±0.01 | 5 | 0.06±0.02 | 10 | 0.02±0.01 |  | 4 | 0.03±0.02 | 46 | 0.04±0.01 | 17 | 1.51±0.35 |  |
| **ABCB6** | 4 | 0.14±0.06 | 7 | 0.64±0.40 | 4 | 0.34±0.06 | 9 | 0.36±0.13 |  | 3 | 0.03±0.01 | 29 | 0.16±0.03 |  |  |  |
| **ABCB7** |  |  | 2 | 0.85±0.39 | 3 | 0.45±0.17 | 7 | 0.20±0.07 |  | 2 | 0.96±0.10 | 49 | 0.33±0.04 | 11 | 0.06±0.01 |  |
| **ABCB8** |  |  | 2 | 0.78±0.43 | 3 | 0.30±0.13 | 6 | 0.19±0.05 |  | 3 | 0.22±0.05 | 38 | 0.11±0.02 | 6 | 0.05±0.01 |  |
| **ABCB10** |  |  | 2 | 0.21±0.12 | 3 | 0.20±0.07 | 5 | 0.15±0.05 |  | 2 | 0.14±0.05 | 42 | 0.24±0.04 |  |  |  |
| **ABCB11** | 4 | 0.03±0.01 | 13 | 0.35±0.19 | 5 | 0.24±0.15 | 10 | 0.36±0.05 |  | 4 | 0.46±0.19 | 65 | 0.32±0.04 | 25 | 0.37±0.05 |  |
| **ABCC2** | 1 | 0.03 | 2 | 1.52±1.51 | 2 | 0.54±0.37 | 7 | 0.38±0.18 |  | 2 | 0.08±0.04 | 42 | 0.07±0.01 | 8 | 0.07±0.05 |  |
| **ABCC3** |  |  | 4 | 0.19±0.11 | 1 | 0.05 | 4 | 0.05±0.02 |  | 3 | 0.06±0.03 | 39 | 0.07±0.01 | 24 | 0.15±0.02 |  |
| **ABCC6** | 4 | 0.13±0.06 | 10 | 0.15±0.08 | 3 | 0.10±0.06 | 11 | 0.07±0.02 |  | 4 | 0.38±0.24 | 66 | 0.33±0.03 | 24 | 0.73±0.06 |  |
| **ABCD1** |  |  | 1 | 0.89 | 2 | 0.04±0.01 | 2 | 0.15±0.08 |  | 1 | 0.24 | 27 | 0.19±0.03 | 3 | 0.13±0.08 |  |
| **ABCD3** | 4 | 0.87±0.29 | 12 | 6.09±2.96 | 6 | 4.56±1.85 | 11 | 4.65±1.44 |  | 4 | 6.18±2.08 | 65 | 7.94±0.72 | 24 | 1.05±0.15 |  |
| **ABCD4** |  |  |  |  |  |  | 3 | 0.06±0.01 |  |  |  | 24 | 0.07±0.01 | 6 | 0.04±0.01 |  |
| **ABCE1** | 4 | 1.15±0.37 | 9 | 0.58±0.24 | 1 | 0.29±0.01 | 7 | 1.79±0.39 |  | 3 | 0.60±0.21 | 24 | 0.19±0.03 | 22 | 0.35±0.07 |  |
| **ABCF1** | 3 | 0.55±0.21 | 2 | 0.28±0.26 |  |  | 7 | 0.25±0.05 |  | 4 | 0.09±0.02 | 3 | 0.03±0.01 | 8 | 0.21±0.06 |  |
| **ABCG2** | 3 | 0.01±0.01 |  |  | 1 | 0.06±0.01 | 1 | 0.02±0.01 |  | 2 | 0.01±0.01 | 0 |  | 24 | 0.84±0.09 |  |

| **Supplementary Table 15:** The number of males and females in each sample set. |
| --- |

| **Sex** | **Adult Livers,**  **Set 1** | **Adult Livers,**  **Set 2** | **Paediatric Livers,**  **Set 1** | **Paediatric Livers,**  **Set 2** | **Biliary Atresia Livers** | **Kidney** | **Intestine** | **Brain** | **Skin** |
| --- | --- | --- | --- | --- | --- | --- | --- | --- | --- |
| Male | 15 | 21 | 13 | 10 | 12 | 16 | 12 | 15 | 1 |
| Female | 12 | 18 | 6 | 13 | 13 | 4 | 4 | 7 | 5 |
| Unknown |  |  | 1 | 1 |  |  |  |  |  |
| **Total** | 27 | 39 | 20 | 24 | 25 | 20 | 16 | 22 | 6 |

**References:**

1 Yan H, Cheng L, Jia R, Yao H, Wu H, Shen Y, Zhang Y, Hao P & Zhang Z (2019) ATP-binding cassette sub-family a member1 gene mutation improves lipid metabolic abnormalities in diabetes mellitus. *Lipids Health Dis* **18**, 103.

2 Guan J-Z, Tamasawa N, Brunham LR, Matsui J, Murakami H, Suda T, Ochiai S, Tsutsui M, Kudou K, Satoh K & Hayden MR (2004) A case of tangier disease with a novel mutation in the c-terminal region of ATP-binding cassette transporter A1. *Am J Med Genet* **130A**, 398–401.

3 Rindler TN, Stockman CA, Filuta AL, Brown KM, Snowball JM, Zhou W, Veldhuizen R, Zink EM, Dautel SE, Clair G, Ansong C, Xu Y, Bridges JP & Whitsett JA (2017) Alveolar injury and regeneration following deletion of ABCA3. *JCI Insight* **2**, e97381.

4 Huang X, Xu J, Shen Y, Zhang L, Xu M, Chen M, Ren J, Zhou L, Gong H & Zhong P (2019) Protein profiling of cerebrospinal fluid from patients undergoing vestibular schwannoma surgery and clinical significance. *Biomed Pharmacother* **116**, 108985.

5 Garces F, Jiang K, Molday LL, Stöhr H, Weber BH, Lyons CJ, Maberley D & Molday RS (2018) Correlating the Expression and Functional Activity of ABCA4 Disease Variants With the Phenotype of Patients With Stargardt Disease. *Investig Opthalmology Vis Sci* **59**, 2305.

6 Fu Y, Hsiao J-HT, Paxinos G, Halliday GM & Kim WS (2014) ABCA5 Regulates Amyloid-β Peptide Production and is Associated with Alzheimer’s Disease Neuropathology. *J Alzheimer’s Dis* **43**, 857–869.

7 Pennings M, Meurs I, Ye D, Out R, Hoekstra M, Van Berkel TJC & Eck M Van (2006) Regulation of cholesterol homeostasis in macrophages and consequences for atherosclerotic lesion development. *FEBS Lett* **580**, 5588–5596.

8 Hollingworth P, Harold D, Sims R, Gerrish A, Lambert J-C, Carrasquillo MM, Abraham R, Hamshere ML, Pahwa JS, Moskvina V, Dowzell K, Jones N, Stretton A, Thomas C, Richards A, Ivanov D, Widdowson C, Chapman J, Lovestone S, Powell J, Proitsi P, Lupton MK, Brayne C, Rubinsztein DC, Gill M, Lawlor B, Lynch A, Brown KS, Passmore PA, Craig D, McGuinness B, Todd S, Holmes C, Mann D, Smith AD, Beaumont H, Warden D, Wilcock G, Love S, Kehoe PG, Hooper NM, Vardy ERLC, Hardy J, Mead S, Fox NC, Rossor M, Collinge J, Maier W, Jessen F, Rüther E, Schürmann B, Heun R, Kölsch H, van den Bussche H, Heuser I, Kornhuber J, Wiltfang J, Dichgans M, Frölich L, Hampel H, Gallacher J, Hüll M, Rujescu D, Giegling I, Goate AM, Kauwe JSK, Cruchaga C, Nowotny P, Morris JC, Mayo K, Sleegers K, Bettens K, Engelborghs S, De Deyn PP, Van Broeckhoven C, Livingston G, Bass NJ, Gurling H, McQuillin A, Gwilliam R, Deloukas P, Al-Chalabi A, Shaw CE, Tsolaki M, Singleton AB, Guerreiro R, Mühleisen TW, Nöthen MM, Moebus S, Jöckel K-H, Klopp N, Wichmann H-E, Pankratz VS, Sando SB, Aasly JO, Barcikowska M, Wszolek ZK, Dickson DW, Graff-Radford NR, Petersen RC, van Duijn CM, Breteler MMB, Ikram MA, DeStefano AL, Fitzpatrick AL, Lopez O, Launer LJ, Seshadri S, Berr C, Campion D, Epelbaum J, Dartigues J-F, Tzourio C, Alpérovitch A, Lathrop M, Feulner TM, Friedrich P, Riehle C, Krawczak M, Schreiber S, Mayhaus M, Nicolhaus S, Wagenpfeil S, Steinberg S, Stefansson H, Stefansson K, Snædal J, Björnsson S, Jonsson P V, Chouraki V, Genier-Boley B, Hiltunen M, Soininen H, Combarros O, Zelenika D, Delepine M, Bullido MJ, Pasquier F, Mateo I, Frank-Garcia A, Porcellini E, Hanon O, Coto E, Alvarez V, Bosco P, Siciliano G, Mancuso M, Panza F, Solfrizzi V, Nacmias B, Sorbi S, Bossù P, Piccardi P, Arosio B, Annoni G, Seripa D, Pilotto A, Scarpini E, Galimberti D, Brice A, Hannequin D, Licastro F, Jones L, Holmans PA, Jonsson T, Riemenschneider M, Morgan K, Younkin SG, Owen MJ, O’Donovan M, Amouyel P & Williams J (2011) Common variants at ABCA7, MS4A6A/MS4A4E, EPHA1, CD33 and CD2AP are associated with Alzheimer’s disease. *Nat Genet* **43**, 429–435.

9 Trigueros-Motos L, van Capelleveen JC, Torta F, Castaño D, Zhang L-H, Chai EC, Kang M, Dimova LG, Schimmel AWM, Tietjen I, Radomski C, Tan LJ, Thiam CH, Narayanaswamy P, Wu DH, Dorninger F, Yakala GK, Barhdadi A, Angeli V, Dubé M-P, Berger J, Dallinga-Thie GM, Tietge UJF, Wenk MR, Hayden MR, Hovingh GK & Singaraja RR (2017) ABCA8 Regulates Cholesterol Efflux and High-Density Lipoprotein Cholesterol Levels. *Arterioscler Thromb Vasc Biol* **37**, 2147–2155.

10 Bleasel JM, Wong JH, Halliday GM & Kim WS (2014) Lipid dysfunction and pathogenesis of multiple system atrophy. *Acta Neuropathol Commun* **2**, 15.

11 Piehler A, Kaminski WE, Wenzel JJ, Langmann T & Schmitz G (2002) Molecular structure of a novel cholesterol-responsive A subclass ABC transporter, ABCA9. *Biochem Biophys Res Commun* **295**, 408–416.

12 Hedditch EL, Gao B, Russell AJ, Lu Y, Emmanuel C, Beesley J, Johnatty SE, Chen X, Harnett P, George J, Williams RT, Flemming C, Lambrechts D, Despierre E, Lambrechts S, Vergote I, Karlan B, Lester J, Orsulic S, Walsh C, Fasching P, Beckmann MW, Ekici AB, Hein A, Matsuo K, Hosono S, Nakanishi T, Yatabe Y, Pejovic T, Bean Y, Heitz F, Harter P, du Bois A, Schwaab I, Hogdall E, Kjaer SK, Jensen A, Hogdall C, Lundvall L, Engelholm SA, Brown B, Flanagan J, Metcalf MD, Siddiqui N, Sellers T, Fridley B, Cunningham J, Schildkraut J, Iversen E, Weber RP, Berchuck A, Goode E, Bowtell DD, Chenevix-Trench G, DeFazio A, Norris MD, MacGregor S, Haber M & Henderson MJ (2014) ABCA Transporter Gene Expression and Poor Outcome in Epithelial Ovarian Cancer. *JNCI J Natl Cancer Inst* **106**, dju149.

13 Wenzel JJ, Kaminski WE, Piehler A, Heimerl S, Langmann T & Schmitz G (2003) ABCA10, a novel cholesterol-regulated ABCA6-like ABC transporter. *Biochem Biophys Res Commun* **306**, 1089–1098.

14 Kaminski WE, Piehler A & Wenzel JJ (2006) ABC A-subfamily Transporters: Structure, Function and Disease. *Biochim Biophys Acta - Mol Basis Dis* **1762**, 510–524.

15 Glavinas H, Krajcsi P, Cserepes J & Sarkadi B (2004) The Role of ABC Transporters in Drug Resistance, Metabolism and Toxicity. *Curr Drug Deliv* **1**, 27–42.

16 Lefevre C (2003) Mutations in the transporter ABCA12 are associated with lamellar ichthyosis type 2. *Hum Mol Genet* **12**, 2369–2378.

17 Tomioka M, Toda Y, Kurisu J, Kimura Y, Kengaku M & Ueda K (2012) The Effects of Neurological Disorder-Related Codon Variations of ABCA13 on the Function of the ABC Protein. *Biosci Biotechnol Biochem* **76**, 2289–2293.

18 Han L, Zuo B, Cai W, Guo Z, Tong B, Wei H, Zhu Z & Li G (2020) Association between ABCB1 (3435C>T) polymorphism and susceptibility of colorectal cancer: A meta-analysis. *Medicine (Baltimore)* **99**, e19189.

19 Abele R & Tampé R (2018) Moving the Cellular Peptidome by Transporters. *Front Cell Dev Biol* **6**, 43.

20 Zhao Y, Ishigami M, Nagao K, Hanada K, Kono N, Arai H, Matsuo M, Kioka N & Ueda K (2015) ABCB4 exports phosphatidylcholine in a sphingomyelin-dependent manner. *J Lipid Res* **56**, 644–652.

21 Zheng M, Zhang H, Dill DL, Clark JD, Tu S, Yablonovitch AL, Tan MH, Zhang R, Rujescu D, Wu M, Tessarollo L, Vieira W, Gottesman MM, Deng S, Eberlin LS, Zare RN, Billard J-M, Gillet J-P, Li JB & Peltz G (2015) The Role of Abcb5 Alleles in Susceptibility to Haloperidol-Induced Toxicity in Mice and Humans. *PLOS Med* **12**, e1001782.

22 Issitt T, Bosseboeuf E, De Winter N, Dufton N, Gestri G, Senatore V, Chikh A, Randi AM & Raimondi C (2019) Neuropilin-1 Controls Endothelial Homeostasis by Regulating Mitochondrial Function and Iron-Dependent Oxidative Stress. *iScience* **11**, 205–223.

23 Fukuda Y, Cheong PL, Lynch J, Brighton C, Frase S, Kargas V, Rampersaud E, Wang Y, Sankaran VG, Yu B, Ney PA, Weiss MJ, Vogel P, Bond PJ, Ford RC, Trent RJ & Schuetz JD (2016) The severity of hereditary porphyria is modulated by the porphyrin exporter and Lan antigen ABCB6. *Nat Commun* **7**, 12353.

24 Miyake A, Higashijima S, Kobayashi D, Narita T, Jindo T, Setiamarga DHE, Ohisa S, Orihara N, Hibiya K, Konno S, Sakaguchi S, Horie K, Imai Y, Naruse K, Kudo A & Takeda H (2008) Mutation in the abcb7 gene causes abnormal iron and fatty acid metabolism in developing medaka fish. *Dev Growth Differ* **50**, 703–716.

25 Nikpour M, Scharenberg C, Liu A, Conte S, Karimi M, Mortera-Blanco T, Giai V, Fernandez-Mercado M, Papaemmanuil E, Högstrand K, Jansson M, Vedin I, Stephen Wainscoat J, Campbell P, Cazzola M, Boultwood J, Grandien A & Hellström-Lindberg E (2013) The transporter ABCB7 is a mediator of the phenotype of acquired refractory anemia with ring sideroblasts. *Leukemia* **27**, 889–896.

26 Ardehali H, O’Rourke B & Marbán E (2005) Cardioprotective Role of the Mitochondrial ATP-Binding Cassette Protein 1. *Circ Res* **97**, 740–742.

27 Basu R, Baumgaertel N, Wu S & Kopchick JJ (2017) Growth Hormone Receptor Knockdown Sensitizes Human Melanoma Cells to Chemotherapy by Attenuating Expression of ABC Drug Efflux Pumps. *Horm Cancer* **8**, 143–156.

28 Fu Z, Liu F, Liu C, Jin B, Jiang Y, Tang M, Qi X & Guo X (2019) Mutant huntingtin inhibits the mitochondrial unfolded protein response by impairing ABCB10 mRNA stability. *Biochim Biophys acta Mol basis Dis* **1865**, 1428–1435.

29 Seguin A, Takahashi-Makise N, Yien YY, Huston NC, Whitman JC, Musso G, Wallace JA, Bradley T, Bergonia HA, Kafina MD, Matsumoto M, Igarashi K, Phillips JD, Paw BH, Kaplan J & Ward DM (2017) Reductions in the mitochondrial ABC transporter Abcb10 affect the transcriptional profile of heme biosynthesis genes. *J Biol Chem* **292**, 16284–16299.

30 Huynh MT, Nguyen T-T, Grison S, Lascols O, Fernandez E & Barbu V (2019) Clinical characteristics and genetic profiles of young and adult patients with cholestatic liver disease. *Rev Española Enfermedades Dig* **111**, 775–788.

31 Carew MW, Naranmandura H, Shukalek CB, Le XC & Leslie EM (2011) Monomethylarsenic Diglutathione Transport by the Human Multidrug Resistance Protein 1 (MRP1/ ABCC1 ). *Drug Metab Dispos* **39**, 2298–2304.

32 Schuetz JD, Swaan PW & Tweedie DJ (2014) The role of transporters in toxicity and disease. *Drug Metab Dispos* **42**, 541–5.

33 Nies AT, König J, Pfannschmidt M, Klar E, Hofmann WJ & Keppler D (2001) Expression of the multidrug resistance proteins MRP2 and MRP3 in human hepatocellular carcinoma. *Int J cancer* **94**, 492–9.

34 Young LC, Campling BG, Cole SPC, Deeley RG & Gerlach JH (2001) Multidrug resistance proteins MRP3, MRP1, and MRP2 in lung cancer: Correlation of protein levels with drug response and messenger RNA levels. *Clin Cancer Res* **7**, 1798–804.

35 Wang J, Zhang M, Zhang L, Cai H, Zhou S, Zhang J & Wang Y (2010) Correlation of Nrf2, HO-1, and MRP3 in Gallbladder Cancer and Their Relationships to Clinicopathologic Features and Survival. *J Surg Res* **164**, e99–e105.

36 Jin D, Ni TT, Sun J, Wan H, Amack JD, Yu G, Fleming J, Chiang C, Li W, Papierniak A, Cheepala S, Conseil G, Cole SPC, Zhou B, Drummond IA, Schuetz JD, Malicki J & Zhong TP (2014) Prostaglandin signalling regulates ciliogenesis by modulating intraflagellar transport. *Nat Cell Biol* **16**, 841–51.

37 Ho LL, Kench JG, Handelsman DJ, Scheffer GL, Stricker PD, Grygiel JG, Sutherland RL, Henshall SM, Allen JD & Horvath LG (2008) Androgen regulation of multidrug resistance-associated protein 4 (MRP4/ABCC4) in prostate cancer. *Prostate* **68**, 1421–1429.

38 Copsel S, Garcia C, Diez F, Vermeulem M, Baldi A, Bianciotti LG, Russel FGM, Shayo C & Davio C (2011) Multidrug Resistance Protein 4 (MRP4/ABCC4) Regulates cAMP Cellular Levels and Controls Human Leukemia Cell Proliferation and Differentiation. *J Biol Chem* **286**, 6979–6988.

39 Holla VR, Backlund MG, Yang P, Newman RA & DuBois RN (2008) Regulation of Prostaglandin Transporters in Colorectal Neoplasia. *Cancer Prev Res* **1**, 93–99.

40 Wijnholds J, Mol CAAM, van Deemter L, de Haas M, Scheffer GL, Baas F, Beijnen JH, Scheper RJ, Hatse S, De Clercq E, Balzarini J & Borst P (2000) Multidrug-resistance protein 5 is a multispecific organic anion transporter able to transport nucleotide analogs. *Proc Natl Acad Sci* **97**, 7476–7481.

41 Ilias A, Urban Z, Seidl TL, Le Saux O, Sinko E, Boyd CD, Sarkadi B & Varadi A (2002) Loss of ATP-dependent Transport Activity in Pseudoxanthoma Elasticum-associated Mutants of Human ABCC6 (MRP6). *J Biol Chem* **277**, 16860–16867.

42 Contrò G, Tallerico R, Dattilo V, Fabiani F, Enzo MV, Hladnik U, Dastoli S, Nisticò SP, Colao E, Perrotti N & Iuliano R (2019) A novel ABCC6 variant causative of pseudoxanthoma elasticum. *Hum Genome Var* **6**, 30.

43 Shah VS, Meyerholz DK, Tang XX, Reznikov L, Abou Alaiwa M, Ernst SE, Karp PH, Wohlford-Lenane CL, Heilmann KP, Leidinger MR, Allen PD, Zabner J, McCray PB, Ostedgaard LS, Stoltz DA, Randak CO & Welsh MJ (2016) Airway acidification initiates host defense abnormalities in cystic fibrosis mice. *Science* **351**, 503–7.

44 Ousingsawat J, Kongsuphol P, Schreiber R & Kunzelmann K (2011) CFTR and TMEM16A are Separate but Functionally Related Cl^-^ Channels. *Cell Physiol Biochem* **28**, 715–724.

45 Saint-Martin C, Zhou Q, Martin GM, Vaury C, Leroy G, Arnoux J-B, de Lonlay P, Shyng S-L & Bellanné-Chantelot C (2015) Monoallelic ABCC8 mutations are a common cause of diazoxide-unresponsive diffuse form of congenital hyperinsulinism. *Clin Genet* **87**, 448–454.

46 Babenko AP, Gonzalez G, Aguilar-Bryan L & Bryan J (1998) Reconstituted Human Cardiac K ATP Channels. *Circ Res* **83**, 1132–1143.

47 Allebrandt K V., Amin N, Müller-Myhsok B, Esko T, Teder-Laving M, Azevedo RVDM, Hayward C, van Mill J, Vogelzangs N, Green EW, Melville SA, Lichtner P, Wichmann H-E, Oostra BA, Janssens ACJW, Campbell H, Wilson JF, Hicks AA, Pramstaller PP, Dogas Z, Rudan I, Merrow M, Penninx B, Kyriacou CP, Metspalu A, van Duijn CM, Meitinger T & Roenneberg T (2013) A KATP channel gene effect on sleep duration: from genome-wide association studies to function in Drosophila. *Mol Psychiatry* **18**, 122–132.

48 Hopper-Borge E, Chen Z-S, Shchaveleva I, Belinsky MG & Kruh GD (2004) Analysis of the Drug Resistance Profile of Multidrug Resistance Protein 7 (ABCC10). *Cancer Res* **64**, 4927–4930.

49 Chen Z-S, Hopper-Borge E, Belinsky MG, Shchaveleva I, Kotova E & Kruh GD (2003) Characterization of the Transport Properties of Human Multidrug Resistance Protein 7 (MRP7, ABCC10). *Mol Pharmacol* **63**, 351–358.

50 Guo Y, Kotova E, Chen Z-S, Lee K, Hopper-Borge E, Belinsky MG & Kruh GD (2003) MRP8, ATP-binding Cassette C11 (ABCC11), Is a Cyclic Nucleotide Efflux Pump and a Resistance Factor for Fluoropyrimidines 2′,3′-Dideoxycytidine and 9′-(2′-Phosphonylmethoxyethyl)adenine. *J Biol Chem* **278**, 29509–29514.

51 Chen Z-S, Guo Y, Belinsky MG, Kotova E & Kruh GD (2005) Transport of Bile Acids, Sulfated Steroids, Estradiol 17-β-d-Glucuronide, and Leukotriene C4 by Human Multidrug Resistance Protein 8 (ABCC11). *Mol Pharmacol* **67**, 545–557.

52 Tammur J, Prades C, Arnould I, Rzhetsky A, Hutchinson A, Adachi M, Schuetz JD, Swoboda KJ, Ptácek LJ, Rosier M, Dean M & Allikmets R (2001) Two new genes from the human ATP-binding cassette transporter superfamily, ABCC11 and ABCC12, tandemly duplicated on chromosome 16q12. *Gene* **273**, 89–96.

53 Guimarães CP, Sá-Miranda C & Azevedo JE (2005) Probing substrate-induced conformational alterations in adrenoleukodystrophy protein by proteolysis. *J Hum Genet* **50**, 99–105.

54 Kemp S, Theodoulou FL & Wanders RJA (2011) Mammalian peroxisomal ABC transporters: from endogenous substrates to pathology and clinical significance. *Br J Pharmacol* **164**, 1753–1766.

55 Ferdinandusse S, Jimenez-Sanchez G, Koster J, Denis S, Van Roermund CW, Silva-Zolezzi I, Moser AB, Visser WF, Gulluoglu M, Durmaz O, Demirkol M, Waterham HR, Gökcay G, Wanders RJA & Valle D (2015) A novel bile acid biosynthesis defect due to a deficiency of peroxisomal ABCD3. *Hum Mol Genet* **24**, 361–370.

56 Kawaguchi K & Morita M (2016) ABC Transporter Subfamily D: Distinct Differences in Behavior between ABCD1–3 and ABCD4 in Subcellular Localization, Function, and Human Disease. *Biomed Res Int* **2016**, 1–11.

57 Coelho D, Kim JC, Miousse IR, Fung S, du Moulin M, Buers I, Suormala T, Burda P, Frapolli M, Stucki M, Nürnberg P, Thiele H, Robenek H, Höhne W, Longo N, Pasquali M, Mengel E, Watkins D, Shoubridge EA, Majewski J, Rosenblatt DS, Fowler B, Rutsch F & Baumgartner MR (2012) Mutations in ABCD4 cause a new inborn error of vitamin B12 metabolism. *Nat Genet* **44**, 1152–1155.

58 Kawaguchi K, Okamoto T, Morita M & Imanaka T (2016) Translocation of the ABC transporter ABCD4 from the endoplasmic reticulum to lysosomes requires the escort protein LMBD1. *Sci Rep* **6**, 30183.

59 Anderson DE, Pfeffermann K, Kim SY, Sawatsky B, Pearson J, Kovtun M, Corcoran DL, Krebs Y, Sigmundsson K, Jamison SF, Yeo ZZJ, Rennick LJ, Wang L-F, Talbot PJ, Duprex WP, Garcia-Blanco MA & von Messling V (2019) Comparative Loss-of-Function Screens Reveal ABCE1 as an Essential Cellular Host Factor for Efficient Translation of Paramyxoviridae and Pneumoviridae. *MBio* **10**, e00826-19.

60 Wilcox SM, Arora H, Munro L, Xin J, Fenninger F, Johnson LA, Pfeifer CG, Choi KB, Hou J, Hoodless PA & Jefferies WA (2017) The role of the innate immune response regulatory gene ABCF1 in mammalian embryogenesis and development. *PLoS One* **12**, e0175918.

61 Bao L, Wu J, Dodson M, Rojo de la Vega EM, Ning Y, Zhang Z, Yao M, Zhang DD, Xu C & Yi X (2017) ABCF2 , an Nrf2 target gene, contributes to cisplatin resistance in ovarian cancer cells. *Mol Carcinog* **56**, 1543–1553.

62 Courtney SC, Di H, Stockman BM, Liu H, Scherbik S V. & Brinton MA (2012) Identification of Novel Host Cell Binding Partners of Oas1b, the Protein Conferring Resistance to Flavivirus-Induced Disease in Mice. *J Virol* **86**, 7953–7963.

63 Wang N, Ranalletta M, Matsuura F, Peng F & Tall AR (2006) LXR-Induced Redistribution of ABCG1 to Plasma Membrane in Macrophages Enhances Cholesterol Mass Efflux to HDL. *Arterioscler Thromb Vasc Biol* **26**, 1310–1316.

64 Toyoda Y, Takada T & Suzuki H (2019) Inhibitors of Human ABCG2: From Technical Background to Recent Updates With Clinical Implications. *Front Pharmacol* **10**, 208.

65 Dodacki A, Wortman M, Saubaméa B, Chasseigneaux S, Nicolic S, Prince N, Lochus M, Raveu A-L, Declèves X, Scherrmann J-M, Patel SB & Bourasset F (2017) Expression and function of Abcg4 in the mouse blood-brain barrier: role in restricting the brain entry of amyloid-β peptide. *Sci Rep* **7**, 13393.

66 Lee J-Y, Kinch LN, Borek DM, Wang J, Wang J, Urbatsch IL, Xie X-S, Grishin N V., Cohen JC, Otwinowski Z, Hobbs HH & Rosenbaum DM (2016) Crystal structure of the human sterol transporter ABCG5/ABCG8. *Nature* **533**, 561–564.

67 Renner O, Lütjohann D, Richter D, Strohmeyer A, Schimmel S, Müller O, Stange EF & Harsch S (2013) Role of the ABCG8 19H risk allele in cholesterol absorption and gallstone disease. *BMC Gastroenterol* **13**, 30.
